# A Psychological Exploration of Engagement in Geek Culture

**DOI:** 10.1371/journal.pone.0142200

**Published:** 2015-11-18

**Authors:** Jessica McCain, Brittany Gentile, W. Keith Campbell

**Affiliations:** 1 Department of Psychology, University of Georgia, Athens, Georgia, United States of America; 2 Outcomes Research, ICON plc, San Francisco, California, United States of America; Medical University of Vienna, AUSTRIA

## Abstract

Geek culture is a subculture of enthusiasts that is traditionally associated with obscure media (Japanese animation, science fiction, video games, etc.). However, geek culture is becoming increasingly mainstream; for example, in the past year alone, Dragon*Con, a major Geek convention in Atlanta, Georgia, attracted an attendance of over 57,000 members. The present article uses an individual differences approach to examine three theoretical accounts of geek culture. Seven studies (N = 2354) develop the Geek Culture Engagement Scale (GCES) to quantify geek engagement and assess its relationships to theoretically relevant personality and individual differences variables. These studies present evidence that individuals may engage in geek culture in order to maintain narcissistic self-views (the great fantasy migration hypothesis), to fulfill belongingness needs (the belongingness hypothesis), and to satisfy needs for creative expression (the need for engagement hypothesis). Geek engagement is found to be associated with elevated grandiose narcissism, extraversion, openness to experience, depression, and subjective well-being across multiple samples. These data lay the groundwork for further exploration of geek culture as well as provide a foundation for examining other forms of subculture participation.

## Introduction

A geek is traditionally defined as an enthusiast who develops expertise on a topic through exceptional determination and devotion [1]. The word “geek” is used to describe not only enthusiasts in science, technology, and engineering but also especially devoted fans of media (i.e., “fandom geeks”). Here, we refer to geek culture as a subculture of enthusiasts that is traditionally associated with obscure media (Japanese animation, science fiction, video games, etc.). However, geek culture is becoming an increasingly mainstream influence on contemporary culture. Geek culture includes a range of activities such as role-playing games (e.g., *Dungeons and Dragons*), science fiction (e.g., *Star Trek*), comic books, and dressing in costumes (i.e., cosplay). Although geek interests were once marginalized [2], comic book movie adaptations (e.g., *Iron Man*, *Thor*) [3] are now major box office draws. Likewise, science-fiction (sci-fi) and fantasy themed video games (e.g., *World of Warcraft*) have become multi-billion dollar industries. There has also been enormous growth in geek conventions such as Comic-con and Dragon*Con. In the past year alone, New York Comic-Con, one of the premier geek conventions in the United States, attracted over 130,000 attendees [4] and Dragon*Con in Atlanta has grown from 2,400 fans in 1989 to 57,000 fans in 2013 [5].

Despite the popularity of geek culture, it has received little attention from the social sciences. Yet this increasing tendency of individuals to engage in a culture with heroic and magical themes may be linked to important trends in our wider culture, such as increasing narcissism[6], thwarted belongingness [7], and the interface between technology and entertainment media. In the present paper, we have two primary goals. First, we develop and validate the construct of geek engagement as participation in specific activities represented at major geek conventions. Second, we describe and examine three new theoretical accounts of geek culture related to the cultural trends above, which we refer to as the *great fantasy migration hypothesis*, the *belongingness hypothesis*, and the *desire for engagement hypothesis*. These theoretical accounts are not considered to be mutually exclusive—participation in geek culture is almost certainly determined by multiple factors and several of these hypotheses share predictions. This research is designed to be the first rather than last word on these hypotheses.

To these ends, we present results from 7 studies (N = 2354). These include construct operationalization and scale development (Studies 1–2), and examination of personality, self-concept, intelligence and other individual differences variables associated with geek engagement as well as a social network analysis of geek culture (Studies 2–7). Before we begin, however, we (a) define geek culture, and (b) describe three theoretical accounts.

### What is Geek Culture?

According to a wide ranging review [2], as early as the 1950’s, the term “geek” and the similar term “nerd” had been used to denote social outcasts in grade schools. Nerds were considered to be socially awkward and overly intellectual, whereas geeks were prone to obsessive interest in marginalized or obscure hobbies such as the *Dungeons and Dragons* game, comic books, and personal computing. These definitions of “geek” and “nerd,” while common, are by no means official, and use of these terms varies between sources. For the sake of simplicity, we will be using only the term geek in this paper to refer to obscure media enthusiasts. Both “geek” and “nerd” were pejorative terms until the 1980’s, when the growing popularity of technology and computers made these former outcasts increasingly useful to society [2]. During this time, geeks began adopting the term for themselves to express pride in their membership in a media and computer-based subculture. A canonical list of media interests that were geeky began to form, including science-fiction and fantasy, comic books, roleplaying games, costuming, etc. These interests tended to share common themes, such as larger-than-life fantasy worlds (e.g., Tolkein’s *Middle Earth*), characters with extraordinary abilities (e.g., Superman), the use of magic or highly advanced technologies (e.g., futuristic technologies in *Star Trek*), and elements from history (e.g., renaissance fairs) or foreign cultures (e.g., Japanese cartoons, or *anime*). Demonstrating knowledge of or devotion to these interests became a form of social currency between self-proclaimed geeks [8].

This identification with a set of media interests can be most clearly observed in geek conventions such as Comic-Con. These conventions provide a gathering space where attendees can attend panels, buy merchandise, and wear costumes to show their devotion to a particular show or comic book character. Historically, specific geek interests were too small to independently support a large convention, so at their inception geek conventions sought to include the full spectrum of topics that might be of interest to geeks [8]. Broad inclusion at these conferences had three interesting outcomes. First, it created a broad geek culture rather than only specific subcultures. Second, it prompted cross-pollination across geek interests; for example, at the Dragon*Con parade you might find a zombie storm trooper, mixing Star Wars and Zombie genres. Finally, and directly germane to the present research, the list of interests included in a large geek convention can be considered a sample of canonical geek interests. Thus, one way to operationalize a person’s involvement in geek culture may be to quantify their involvement in each of the geek interests represented at a geek convention. Although this approach may miss some of the more marginal geek interests that are not represented at a geek convention, it provides us with a list of interests that geeks themselves have identified to be geeky.

Based on the information above, we have defined geek culture engagement as a first step to understanding why individuals engage in geek culture. Below we describe three original hypotheses that may help to explain individual geek engagement.

### Theoretical Accounts of Participation in Geek Culture

Although geek culture has been the subject of little psychological study, anthropologists and communications researchers have begun to describe geek culture and provide several theoretical accounts of its widespread appeal [1,2,8–11]. Based on these theories, as well as several from the psychology literature, we have generated three hypotheses. The present data only speaks to why an individual may choose to participate in geek culture. Further research is needed to understand why geek culture is becoming increasingly prominent in contemporary American culture. Please note that it is not our intention to link geek culture with psychiatric dysfunction or antisocial behavior. We have conducted no clinical assessments of any kind. Our theoretical accounts refer to variations in normal personality traits that are not necessarily maladaptive and may even be adaptive in some contexts. Our aim is simply to describe and understand individual motivations for participating in geek culture.

#### The “Great Fantasy Migration” Hypothesis

In US society, inflated self-esteem and narcissism—which have increased steadily over the past few generations—are being met with a harsh reality of low youth employment and high debt loads [6,12,13]. Separate from Narcissistic Personality Disorder (NPD), narcissism is a normal personality trait characterized by a grandiose sense of self as well as efforts to maintain that sense of self in the face of reality [14]. Narcissists can be charismatic [15], confident [16], or even emerge as effective leaders [17], but when faced with failure or criticism, narcissists tend to protect their sense of self through such strategies as discrediting the source of the criticism [18] and withdrawing from challenging tasks in favor of easier routes to self-enhancement [19]. In the United States, narcissism has been increasing since the 1970’s, while traditional ways of supporting narcissism such as prestigious jobs and credit (e.g., the debt bubble collapse) are becoming less viable for the majority of Americans. The result for individuals is discomfort (or cognitive dissonance [20]) with the incongruence between inflated sense of self and deflated reality [13]. One solution for resolving this dissonance is to migrate into a fantasy world via role playing games, fandoms, and fantasy media. These hobbies present opportunities for living out a grandiose self (e.g., by role playing a powerful or charismatic character) that might not be possible in the non-fantasy world. And, of course, in some cases success in fantasy (e.g., tournament gaming, achieving cosplay fame) can lead to real world success. In addition, it is easier to obtain expert status and admiration for one’s knowledge of geek subjects (e.g., *Star Trek* trivia) because credentials such as education and certification are not required. Thus, narcissistic individuals who are unable to receive the admiration and praise to which they feel entitled (whether because of failure, or because their grandiose fantasy is impossible to live out in the real world) may turn to a fantasy world where such praise is more easily obtained.

If the great fantasy migration hypothesis is correct, we should see a correlation between narcissism and participation in geek culture, and perhaps more strongly to the more roleplaying and immersive elements of geek culture. We should also see higher levels of fantasy proneness, a personality trait associated with elevated fantasizing and magical beliefs [21], among individuals engaged in geek culture. Fantasy proneness can be defined as a tendency to have intense daydreams, to have difficulty distinguishing between fantasy and reality, and to have magical or pseudoscientific beliefs. Although fantasy proneness has typically been associated with dysfunction, recent work has shown it to have two factors: a factor associated with strong imagery and strange beliefs and a factor associated with daydreaming and enjoyment of fantasy [22]. While the former factor is associated with dysfunction, the latter is not. Thus, normally occurring levels of fantasy proneness may positively predict geek engagement even in normally functioning individuals. Finally, to the extent that individuals participate in geek culture we should see reduced civic engagement and less engagement in real life goals, such as career aspirations and raising a family, as these would likely be less viable sources of praise and esteem and thus would be less rewarding to narcissists than geek culture activities.

#### The Belongingness Hypothesis

Belongingness, or the desire to form and maintain stable interpersonal relationships, is theorized to be a basic human need [23]. Self-Determination Theory (SDT) [24] lists relatedness (an equivalent construct) as one of the three basic needs that motivate human behavior, implying that much of an individual’s choices and interests in life will be in service to this need. Leary et al. [25] propose that self-esteem is contingent upon acceptance from others, and Social Identity Theory [26] states that individuals seek to join and identify with groups (such as fan groups [27]) to maintain that self-esteem. Thus engagement in geek culture may be distinguished by the particular strategy of using common media interests to fulfill needs for belongingness.

The above statement is consistent with anthropological work on geeks. Woo [9] characterized geek culture as a way of creating community in an increasingly individualistic society. Because traditional resources for fulfilling belongingness needs such as civic groups, the nuclear family, and strong communities have weakened or all but disappeared for the current generation [7], Woo proposed that geeks gain belongingness by rallying around the resources that are currently available: consumer goods and cultural artifacts. Woo’s hypotheses were supported by his finding that geeks use knowledge of geek interests (e.g., *Star Trek* trivia) and collections (e.g., model spaceships) as social currency [8]. Along the same vein, Tocci [2] described a process by which people who are outcast or rejected as children devote more energy to exploring solitary interests, including obscure interests, and eventually form ties to others with the same specialized interests, thereby forming a network of relationships based around previously solitary activities. He theorizes that the internet has amplified this process by providing increased access to information on obscure interests as well as a way to connect anonymously with others who share those interests. Via the internet, individuals who have rare and unusual interests can more easily find and contact each other as well as recruit new enthusiasts. These sources suggest that geek culture may provide a path to fulfilling belongingness that is more accessible for certain individuals because it is based on (previously) solitary interests and hobbies and uses one’s devotion to them as social capital.

If the belongingness hypothesis is correct, we can expect that participants will report greater positive self-feelings when engaging in activities they believe others will accept them for, in keeping with past research on belongingness [25]. We also expect individuals who expect greater acceptance from important others when engaging in geek activities to identify more strongly as a geek, consistent with Social Identity Theory. We can also expect that those with higher levels of geek engagement will report closer associations or ties with others who share those interests. The latter phenomenon is commonly referred to in the social sciences as homophily [28] or in Social Identity Theory as felt closeness to one’s group [26].

#### The Desire for Engagement Hypothesis

Mizer [29] and Konzack [30] see geek culture as a counterculture against a growing power differential in the media. As entertainment becomes monopolized by a few commercial entities and the public is expected to be increasingly passive receptors of media, individuals who identify as geeks seek to actively participate in their entertainment by role-playing, creating fan-fiction, and behaving as though fictional universes are real. Mizer calls the latter activity the “ironic imagination” and describes it as particularly dependent on social interaction, as getting multiple people to treat a fantasy universe as real can extend the escapism beyond the original work of fiction. Consistent with this view, fandom members (who fit the definition of a geek) have been shown to distinguish themselves from more passive media consumers through their agency in shaping media [10], their ability to handle extreme or taboo content [11], and their active intellectual engagement [1,11] with media, at times referring to non-geeks as less intelligent or aware [1,11]. Therefore, geek culture may be distinguished by the tendency to actively participate in one’s own escapism and entertainment, especially in tandem with other people.

It may be that these individuals engage more with media because of a greater need for stimulation, whether intellectual or emotional. Individuals high in certain traits associated with the need for stimulation, such as need for cognition (which refers to the enjoyment of thinking and preference for more complex tasks [31]) and sensation seeking (which refers to the desire for new experiences and novelty [32]) as well as openness to experience (which includes preferences for variety and appreciation of aesthetics [33]) may find active participation in media (such as roleplaying and game playing) and more novel and unusual media content (such as fantasy and science fiction) preferable to mainstream media. If this is true, individuals high in need for cognition and sensation seeking may be more likely to be engaged in geek culture. In addition, creative individuals are known to need stimulation and novelty [34] and a significant portion of geeks’ engagement with media takes place through creative activities (e.g., Anime subbing and fanfiction [35,36]). It may be that individuals high in creativity may also be more likely to engage in geek culture. Finally, because geek media deals mainly with fantasy content, fantasy proneness may make such people even more likely to choose geek culture to fulfill their entertainment needs.

If the desire for engagement hypothesis is correct, we would expect people high in need for cognition, creativity, sensation seeking, and openness to experience to report higher levels of geek engagement. We would also expect intelligence, which is known to have relationships to need for cognition [37] and openness [38,39], to be positively related to geek engagement, consistent with the stereotype that geeks are particularly intelligent. Finally, because fantasy proneness, openness to experience, and adaptive (nondysfunctional) levels of schizotypal personality and dissociation form a constellation of traits that positively predict creativity [21,39–41], we predict that the latter two traits will be associated with geek engagement as well.

### The Present Research

The present research aims to: (a) provide preliminary tests of the above hypotheses by exploring the individual differences and social behaviors associated with geek culture engagement and (b) operationalize geek culture by creating measures of geek culture involvement and identity. All studies (except Study 2) used participants from Amazon’s Mechanical Turk (MTurk), which have been shown to give data of similar quality to traditional samples [42,43]. All studies in this paper were approved and monitored by the Institutional Review Board of the University of Georgia (Approval numbers: Study 1 2013106420; Study 2 STUDY00000229; Study 3 STUDY00000203; Study 4 STUDY00000563; Study 5 STUDY00000273; Study 6 STUDY00000413; Study 7 STUDY00000783). Participants gave informed consent by clicking “I consent” on a consent script in all studies except for Study 2; for Study 2, participants gave written consent by signing a consent form. Studies varied between using general samples and self-described geek samples. Details of all samples are reported in Table 1. Where appropriate, the series mean was imputed for all missing data values in this and all remaining studies. For the majority of questions in all studies less than 1% of values were imputed, and the highest percentage was 3% in Study 4. However, the results of Study 4 did not differ whether or not missing values were imputed.

**Table 1:** Characteristics of Samples 1–8.

		Gender	% Race					Age		Income Per Year (in thousands)		
Study	N	% Male	White	Black	Asian	Hispanic	Mixed Race	Mean	SD	% <$20	% $20-$75	% >$75
1(S A)	350	54	67	9	8	1	12	29.3	8.5	20	60	20
1 (S B)	317	44	78	7	6	1	7	34.5	13.07	23	59	17
2	202	50	88	4	5	4	6	30.2	10.07	19	60	21
3	334	39	73	6	4	0	13	34.6	12.32	23	58	19
4	348	36	71	7	5	0	11	35.9	13.39	24	60	17
5	226	43	77	6	4	0	12	35.3	12.89	17	66	17
6	396	38	83	7	3	1	8	36	18.27	19	56	23
7	181	41	86	8	8	0	11.1	30	8.5	22	61	15

Note: All demographics were determined by self-report (i.e., participants chose which of the available terms best described them.)

*Study 1* employs two samples to develop and validate the Geek Culture Engagement Scale (GCES) and Geek Culture Engagement Scale Short Form (GCES-S). The GCES and/or GCES-S is used in all subsequent studies. Study 1 also examines the relationships between geek engagement and personality traits relevant to all three hypotheses. *Study 2* further explores the measurement of geek engagement by having trained raters as well as naïve coders rate photographs of attendees of a major geek convention (i.e., Dragon*Con), and provides further validation for the GCES-S. *Study 3* examines the great fantasy migration hypothesis by measuring civic engagement among those reporting geek culture interests. *Study 4* examined the belongingness hypothesis. Participants rated each geek interest or activity from the GCES in terms of how they feel others will react to their engagement in that activity, as well as how they feel when they engage in the interest or activity and how often they engage in each activity. Study 4 also develops the Geek Identity Scale (GIS) to test whether self-identifying as a geek is related to geek engagement and belongingness motives. *Study 5* presents a homophily analysis of the egocentric networks of 182 individuals. *Study 6* tests the need for engagement hypotheses by testing the relationship between geek engagement and measures of fantasy proneness and associated traits, IQ, Big Five personality, need for cognition, and sensation seeking. Finally, *Study 7* examines creativity in geek culture.

This manuscript reports results of all (N = 7) studies we have conducted to date using the GCES either here or online at https://osf.io/u25x9/ (i.e., there is no file drawer effect) [44]. Furthermore, data files for all studies are available on the same site.

## Study 1

We first sought to operationalize geek culture by creating a scale that could be used to test our hypotheses. Because geek culture defines itself through identification with media interests, we proposed that geek culture engagement could be operationalized by quantifying participation in the interests and activities present at major geek conventions. We thus combined the activities and genres listed in the programs of the internationally successful multigenre convention, Dragon*Con, and added in non-redundant geek-related activities from two other conventions local to Atlanta (Furry Weekend Atlanta and Frolicon, a science fiction and kink convention) to create a representative sample of geek activities. Based in Atlanta, GA, Dragon*Con drew over 57,000 members in 2013, and offers over 30 different “fan tracks” reflecting the varied interests and niches of geek culture [5]. In two Amazon MTurk samples, we used a listing of these fan tracks as well as several measures of personality and emotional needs to begin to validate the construct of geek culture engagement and explore its nomological network (i.e., the set of lawful relationships that define geek culture in relation to other constructs [45]). In our choice of personality measures, we also began to explore all three theoretical accounts by exploring the relationships between geek engagement and narcissism, Big Five personality, and basic emotional needs.

### Methods

#### Procedure

Samples A (N = 350) and B (N = 317) completed the study online. For Sample A, the Amazon MTurk posting was worded to attract people who are engaged in geek culture and to discourage people without interests in geek culture activities from participating (exact wording for all studies is posted online at https://osf.io/u25x9/). For Sample 2, The MTurk posting was worded as generically as possible so as to recruit participants with a variety of geek engagement levels. Participants indicated their consent by clicking “I consent” on a consent script and completed the measures via an online survey hosting website before being compensated via MTurk. Thirty participants were found to have already participated in Sample A and were removed from Sample B.

#### Materials

To test the nomological network of geek engagement, we included measures we theorized to have relationships with geek engagement along with related traits (e.g., we included all of the Big Five Personality Traits [33], although openness had the most theoretical interest). To this end we included measures of grandiose and vulnerable narcissism and entitlement (predicted by the great fantasy migration hypothesis), and the SDT basic psychological needs (relatedness, predicted by the belongingness hypothesis) [46]. We also included measures of SDT motivational orientation (i.e., how oriented an individual is toward aspects of the environment that encourage autonomy, are controlling, or are under the control of the individual) [47], although we made no specific predictions relating to these measures, and depression and life satisfaction, as these would be negatively related to fulfilled ego or belongingness needs.

To measure geek engagement, we created the *Geek Culture Engagement Scale (GCES)*, by generating a list of 37 geek activities (e.g., cosplay, gaming), interests (e.g., science fiction, fantasy) and lifestyles (e.g., lolita, furry) based on the listing of fan tracks on the Dragon*Con website. We also included the item “your real (daily) life” to explore whether participants who were more involved in geek activities were less involved in daily life. We then asked participants to indicate to what extent they participated in each item on a scale from 1 (*a little*) to 5 (*a lot*). See Table 2 for the specific items assessed by the GCES. See Appendix for the full scale.

**Table 2:** Exploratory Factor Analysis of Geek Engagement.

	Role Playing	Hobbies	Puppetry Robotics	Japanese	Genres	Theater	Life Styles	Horror
**LARP**	**.613**	.348	.298	.208	.035	.105	.344	.168
**Tabletop**	**.835**	.481	.257	.188	.255	.260	.327	.207
Computer Gaming	.351	.045	-.117	.135	.329	.100	.013	.197
**Cosplay**	.507	**.561**	.448	.367	.135	.341	.416	.230
Internet	.139	.067	.016	.222	.203	.189	.103	.210
**Renfaire**	.477	**.741**	.248	.194	.172	.461	.346	.313
**SCA**	.493	**.839**	.476	.203	.057	.309	.436	.218
**Weapons**	.391	**.526**	.400	.179	.113	.044	.353	.274
**Paranormal**	.338	**.549**	.338	.113	.154	.311	.401	.517
**Puppetry**	.416	.597	**.812**	.262	.042	.326	.525	.236
**Robots**	.472	.401	**.592**	.260	.154	.215	.238	.281
**Theater**	.402	.478	.483	.241	.181	**.627**	.284	.312
**Creative Writing**	.239	.293	.117	.232	.215	**.450**	.180	.453
Social Network Sites	.207	.138	-.020	.054	.085	.237	.062	.266
Real Life	.015	-.029	-.184	-.056	.122	-.011	-.202	.122
**Fantasy**	.248	.115	-.193	.313	**.608**	.143	.046	.322
**SciFi**	.220	.067	-.001	.214	**.861**	.198	.005	.228
**Anime**	.204	.134	.073	**.907**	.263	.135	.122	.156
**Manga**	.222	.208	.256	**.855**	.171	.209	.251	.151
Comics	.384	.244	.278	.326	.275	.383	.147	.377
Horror	.214	.224	.122	.176	.228	.249	.285	.717
**Broadway**	.319	.427	.192	.275	.252	**.701**	.268	.319
**Alternative History**	.393	.389	.221	.298	.424	**.482**	.292	.420
Cartoons	.140	.288	.111	.242	.231	.358	.139	.375
**British Series**	.220	.197	-.003	.176	**.516**	.491	.059	.263
**Filking**	.437	**.601**	.469	.294	.149	.503	.458	.252
Cinema	.185	.204	.268	.106	.229	.460	.103	.427
**Joss Whedon Films**	.204	.257	.084	.158	.457	**.508**	.158	.426
**Rocky Horror**	.406	.465	.295	.209	.195	**.635**	.418	.410
Skeptic	.118	.049	-.091	.054	.265	.154	.196	.297
**Lolita**	.379	.441	.362	.280	.046	.210	**.808**	.287
**Gothic**	.341	.325	.148	.156	.114	.281	**.671**	.456
**Furry**	.410	.483	.512	.253	.030	.184	**.759**	.128
**Pagan**	.424	.520	.329	.182	.115	.283	**.719**	.342
**BDSM**	.411	.470	.261	.246	.094	.302	**.721**	.383
**Polyamore**	.443	.522	.420	.150	-.032	.241	**.608**	.240

Extraction Method: Maximum Likelihood; Rotation Method: Promax with Kaiser Normalization.

Note: Bolded items were retained for the final GCES.

The *Narcissistic Personality Inventory* (NPI) [48] is a 40-item nonclinical measure of dimensional narcissism (Samples A and B Cronbach α = .89). For each item, participants choose which of two statements (e.g., “I like to be the center of attention”/ “I prefer to blend in with the crowd”) best describes them. Scores range from 0–40 with higher scores indicating more narcissism.

The *Hypersensitive Narcissism Scale* (HSNS) [49] is a 10-item scale (Sample A α = .73; Sample B α = .80) designed to measure vulnerable narcissism. Items such as “my feelings are easily hurt by ridicule or the slighting remarks of others” are rated on a Likert-type scale from 1 (*not at all like me*) to 5 (*very much like* me). Items range from 0–50 with higher scores indicating more vulnerable narcissism.

The *Rosenberg Self-Esteem Scale* (RSES) [50] is a widely used 10-item measure (Sample A α = .91; Sample B α = .93) of explicit self-esteem. Items such as “on the whole, I am satisfied with myself” are rated on a scale from 1 to 5 with 1 signifying “*this statement does not describe me in the slightest*” and 5 signifying “*this statement describes me perfectly*.”

The *Center for Epidemiological Studies Depression Scale* (CES-D) [51] is a 20-item self-rating inventory (Sample A α = .93; Sample B α = .94) that is widely used in the measure of nonclinical depression symptoms (e.g., “I felt depressed”). Respondents rated a list of symptoms on a scale from 0 (*rarely/none of the time*, *less than 1 day*) to 3 (*most or all of the time*, *5–7 days*) as to how often they have experienced each symptom in the past week.

The *Psychological Entitlement Scale* (PES) [52] is a 9-item measure of generalized entitlement (Samples A and B α = .89), which is one of the central components of narcissism [53], defined as the belief that one deserves better treatment than others. The PES allows for a more targeted assessment of entitlement than the NPI or HSNS [52]. Participants indicated their agreement with items such as “great things should come to me” on a scale from 1 (*strongly disagree*) to 7 (*strongly agree*).

The *Five Factor Model Rating Form* (FFMRF) [54] is a 30-item measure of the Big Five personality traits. Participants indicated their identification with each individual facet of the Big Five traits, including agreeableness (e.g., “straightforwardness”; Sample A α = .67; Sample B α = .70), extraversion (e.g., “gregariousness”; Sample A α = .73; Sample B α = .77), conscientiousness (e.g., “competence”; Sample A α = .80; Sample B α = .82), neuroticism (e.g., “anxiousness”; Sample A α = .78; Sample B α = .81), and openness to experience (e.g., “fantasy”; Sample A α = .67; Sample B α = .66) on a 5-point Likert scale.

The *Diener Satisfaction with Life (SWL) scale* [55] is a 5-item scale (Samples A and B α = .92) of subjective well-being. Participants rated items such as “I am satisfied with my life” on a 7-point scale (1 = *strongly disagree*; 7 = *strongly agree*).

The *General Causality Orientations Scale* (GCOS) [56] is a measure of self-determination in personality [57]. It features 17 vignettes describing hypothetical situations. For each vignette, participants rated the likelihood of their pursuing three possible courses of action on a 7-point scale (1 = *very unlikely*; 7 = *very likely*). These courses of action represent three dimensions of self-determination, autonomy (Sample A α = .84; Sample B α = .87), controlledness (Sample A α = .71; Sample B α = .73), and impersonal (Sample A α = .85; Sample B α = .84).

The *Basic Psychological Needs Scales* (BPNS) [46,58] is a collection of scales measuring the basic motivational needs of autonomy (Sample A α = .76; Sample B α = .75), competence (Sample A α = .76; Sample B α = .77), and relatedness (Samples A and B α = .80) in the workplace, in relationships, and in general.

### Results

#### Factor analyses and scale validation

In order to validate the GCES, we conducted maximum likelihood exploratory factor analysis (Promax rotation) on Sample A, which we then replicated via confirmatory factor analysis in Sample B. An eight factor solution (from which the item “conventions” was removed because it produced a Heywood case [59]) was found to be the best fit for the data (χ^2^ (370) = 612.45, *p* < .001, χ^2^/df = 1.66, CFI = .95, TLI = .92, RMSEA = .04, 90% CI [.04,.05], SRMR = .03). These factors were easily interpretable as clusters of related activities and are shown in Table 2. For example, we named factor 1 “Roleplaying” because it appeared to feature both live action role playing (LARP) games and table top role playing games (e.g., *Dungeons and Dragons*). The items computer/console gaming, internet forums, social networking sites, cartoons, real life, and skepticism failed to load at .4 or greater on a factor, while horror loaded on its own separate factor. Because comic books appeared to crossload on more than one factor, it and the above items were omitted from the scale and from further analyses in this study and all subsequent studies. As “Real life” is not an item intended to measure geek culture, this item has been excluded from analyses in all subsequent studies as well as Sample B of this study. A summary of its relationship with Geek Engagement can be found in the meta-analysis portion of this paper. All seven resulting factors were intercorrelated, *r*s = .14 to .70; all of these correlations were positive, suggesting that geek engagement is rarely limited to one cluster of activities. This seven factor solution was tested via confirmatory factor analysis on Sample 2 (χ^2^ (303) = 684.00, *p* < .001, χ^2^/df = 2.26, CFI = .89, TLI = .87, RMSEA = .06, 90% CI [.06,.07], SRMR = .06) and showed tolerable fit, suggesting that this factor solution is stable. The item Cinema was dropped at this stage because it failed to load significantly on any factor in the CFA solution.

#### Zero order correlations and regressions

To begin to test our theoretical accounts of geek culture, and to further validate geek engagement and its factors as constructs, we explored geek culture’s nomological network—the network of lawful relationships [45] that defines geek engagement in relation to other constructs. To do this, we first calculated a full-scale geek engagement score as the average of the responses for each item in the GCES (Sample A α = .92; Sample B α = .95) and measured its zero order correlations with the other personality measures in Samples A and B. These relationships are shown in Table 3. The subscales of both the BPN scale and the GCOS showed no significant relationships to geek engagement in Sample A, but geek engagement was associated with thwarted autonomy needs, *r*(315) = -.13, 95% CI [-.24,-.02], lower autonomy orientation, *r*(315) = -.25, 95% CI [-.35,-.15], and higher impersonal orientation, *r*(315) = .13, 95% CI [.02,.24] in Sample B. In both samples, geek engagement showed the same pattern of positive correlation with narcissism, vulnerable narcissism, neuroticism, openness to experience, depression, extraversion, and entitlement.

**Table 3:** Correlations between GCES (full and short form) and Personality Measures in Samples 1 and 2.

Sample	NPI	RSE	HSNS	Entitlement	N	E	O	A	C	CESD	SWB
S1	**.29 [.19,.39]**	-.03 [-.14,.07]	**.13 [.02,.23]**	**.19 [.09,.29]**	**.19 [.09,.29]**	**.26 [.16,.36]**	**.30 [.20,.39]**	.02 [-08,.13]	.01 [-10,.11]	**.32 [.23,.41]**	**.12 [.02,.23]**
S2	**.27 [.16,.37]**	**-.15 [-.26,-.04]**	**.16 [.05,.26]**	**.15 [.04,.25]**	**.21 [.11,.32]**	**.21 [.10,.31]**	**.26 [.15,.36]**	.06 [-05,.17]	.00 [-11,.11]	**.33 [.23,.42]**	.08 [-.03,.19]
S1 (Short)	**.30 [.20,.39]**	-.04 [-.14,.07]	**.13 [.03,.24]**	**.24 [.14,.34]**	**.18 [.07,.28]**	**.28 [.18,.37]**	**.25 [.15,.35]**	.01 [-09,.12]	.01 [-09,.12]	**.30 [.20,.39]**	**.14 [.04,.24]**
S2 (Short)	**.25 [.14,.35]**	**-.17 [-.27,-.06]**	**.13 [.02,.23]**	**.14 [.03,.25]**	**.19 [.08,.29]**	**.18 [.07,.29]**	**.19 [.08,.29]**	.04 [-07,.15]	.01 [-10,.12]	**.30 [.19,.40]**	.09 [-.02,.20]
S1 (Specialists Only)	**.28 [.04,.48]**	-.02 [-.26,.22]	.15[-.09,.38]	.14 [-.10,.37]	-.06 [-.30,.18]	.21 [-.03,.43]	-.07 [-.30,.17]	-.17 [-39,.07]	.11 [-13,.34]	.23 [-.01,.44]	.17 [-.08,.39]
S2 (Specialists Only)	.20 [-.02,.39]	-.13 [-.34,.08]	.02 [-.19,.24]	.05 [-.16,.26]	.11 [-.11,.31]	.03 [-.18,.24]	.28 [.07,.46]	**-.25 [-.44,-.04]**	-.04 [-.25,.17]	.16 [-.05,.36]	-.15 [-.35,.06]

Note: 95% Confidence intervals in brackets. Confidence intervals not containing 0 in bold. NPI = Narcissistic Personality Inventory; RSE = Rosenberg Self-Esteem; HSNS = Hypersensitive Narcissism Scale; N = Neuroticism; E = Extraversion; O = Openness to Experience; A = Agreeableness; C = Conscientiousness; CESD = Center for Epidemiological Studies Depression Scale; SWB = Subjective Well Being

Because we conceptualized geek engagement as being elevated or higher when individuals are engaged in multiple geek activities, we wanted to test whether the above pattern of relationships differed for geek “specialists,” or individuals who were strongly interested in only one or two geek activities. We therefore separated individuals who answered 4 or 5 for only one or two activities (Sample A: N = 68; Sample B: N = 86) from the rest of the sample (excluding those who did not endorse geek engagement at all) and reran the correlations (see Table 3). In Sample A, although lack of power caused several relationships to lose significance, the relationships differed little in terms of direction and magnitude, with the exception of the Big Five traits neuroticism, openness, agreeableness and conscientiousness and depression. In Sample B, however, only the relationships between narcissism, self-esteem, and openness to experience remained similar. This suggests that specialist geeks may differ from generalist geeks in important ways and that the GCES as it is used in this paper speaks best toward generalist geeks.

We then conducted a series of multiple regressions in order to control for relevant demographic variables. The first multiple regression analysis predicted geek engagement with age, gender, SES, and all of the personality variables. In Sample A, gender, grandiose narcissism, openness, extraversion, depression, and subjective well-being maintained significance. Their continued significance suggests that these relationships are independent of gender and age. For Sample B, grandiose narcissism, depression, and subjective well-being maintained significance. The second multiple regression analysis predicted geek engagement with age, gender, SES, and all of the SDT variables. Although impersonal causality orientation no longer predicted geek engagement, autonomy causal orientation maintained significance in both samples. The results of these and all further regression analyses in the paper can be found online at https://osf.io/u25x9/. Geek engagement showed a significant correlation to gender in both samples, *rs* = .13-.20, in that males showed significantly higher geek engagement, and showed a negative correlation to age in Sample B, *r*(315) = -.14, 95% CI [-.25,-.03].

The variables loading on each factor were then averaged to produce seven unique subscales for the GCES: Lifestyles (Sample A α = .87; Sample B α = .90), Theater (Sample A α = .79; Sample B α = .82), Hobbies (Sample A α = .81; Sample B α = .87), Puppetry/Robotics (Sample A *r* = .55; Sample B *r* = .70), Japanese (Sample A *r* = .77; Sample B *r* = .81), SciFi/Fantasy (Sample A α = .68; Sample B *r* = .79), and Roleplaying (Sample A *r* = .50; Sample B *r* = .67). The relationships between these factor scores and the personality measures in Samples A and B are shown online at https://osf.io/u25x9/.

#### Testing the great fantasy migration hypothesis

The great fantasy migration hypothesis predicts that individuals high in narcissism will score higher in geek engagement. However, it also posits that narcissism will be particularly related to engagement with the more roleplaying and immersive elements of geek culture, because these provided the greatest opportunities for playing out grandiose fantasy. We classified the following subscales as immersive subscales because each contained activities that have a strong emphasis on playing a role (which would allow someone to self-enhance): Lifestyles (e.g., Lolita), Hobbies (e.g., cosplay), Theater (e.g., theater), Roleplaying (e.g., LARPing), and Puppetry/Robotics (e.g., Puppetry). Grandiose narcissism was positively associated with all five subscales in Sample A (*r*s = .21-.34) and all but Hobbies in Sample B (*r*s = .20-.34), but was also significantly related to Japanese (Sample A: *r* = .13; Sample B *r* = .14) in both samples. Vulnerable narcissism predicted Lifestyles, Theater, and Puppetry/Robotics in Sample A (rs = .13-.14) and Lifestyles and Puppetry/Robotics in Sample B (rs = .13-.14) but also predicted Japanese in both samples (*r*s = .14). In a series of regressions containing all the personality grandiose and vulnerable narcissism, self-esteem, the Big Five personality traits, subjective well-being, and depression) and demographics variables (age, gender, and SES), grandiose narcissism no longer predicted Japanese or Hobbies engagement and vulnerable narcissism no longer predicted any subscale scores. These results suggest that although grandiose narcissism does predict most of the immersive elements of geek culture when controlling for demographics, it does not predict hobbies beyond demographics, and vulnerable narcissism does not predict the immersive elements of geek culture when controlling for demographics.

#### Sample differences

We then tested the differences between geek engagement scores in Sample A (which targeted geeks only) and Sample B (which targeted both geeks and non-geeks). The mean geek engagement score of Sample B (M = 1.91, SD = .72) was significantly lower than the mean of Sample A (M = 2.27, SD = .67; *t*[268] = -8.98, *p* < .001; *d* = .52), suggesting that we were successful in recruiting more people with geek interests in Sample A than in Sample B. We used a series of z-tests to compare the correlations with our personality variables across samples. No significant differences arose, suggesting that the relationships between geek engagement and personality are consistent across multiple samples.

#### A short scale

Because of the growing need for brief or concise measures [60], we created a shortened version of our scale, the *Geek Culture Engagement Scale Short Form* (GCES-S) by taking the two items with the highest loadings on each factor from the Sample A factor analysis. The measure is posted online at https://osf.io/u25x9/. This short measure showed the same general pattern of relationships with the above personality variables in Samples A and B (see Table 3), and showed no significant differences in its correlation with any individual differences variables.

### Discussion

Study 1 created and validated the GCES, while also beginning to test the great fantasy migration and belongingness hypotheses. The GCES and the GCES-S appear to show good reliability and a stable seven-factor structure. Geek culture engagement appears to be a valid construct in that it consistently relates to grandiose narcissism, openness, extraversion, depression, and subjective well-being, showing a stable nomological network. One limitation of the scale arose: although few participants reported high engagement in only one or two geek activities, those few differ from other geeks in terms of Big Five personality traits. Thus caution is recommended when using this scale with geek specialists in the future. In addition, researchers are cautioned that the GCES is not an exhaustive list of geek interests, and might miss some marginal geek interests.

The GCES’s consistent relationships to narcissism, depression, and subjective well-being provide preliminary support for the great fantasy migration hypothesis. In addition, grandiose narcissism is related to those subscales involving immersive elements (with the exception of hobbies), while it is unrelated to the Genres and Japanese subscales (which involve simply consuming fantasy, science-fiction, and Japanese media) after controlling demographics. This is only partially consistent for the great fantasy migration hypothesis. Grandiose narcissism was not related to hobbies, although this may be because although hobbies includes cosplay (a roleplaying element), the majority of hobbies listed do not provide strong opportunities to self-enhance. In addition, vulnerable narcissism was not related to any of the immersive elements of geek culture after controlling for demographics. This implies that only grandiose narcissism is related to fantasy migration.

Finally, the results of Study 1 are inconsistent with the belongingness hypothesis. Although in Sample B the scale showed relationships to measures of Self-Determination Theory [24] consistent with thwarted autonomy needs, there was no correlation to relatedness needs. Thus we failed to provide support for the belongingness hypothesis in this study.

Study 1 established the factor structure and nomological network of the GCES. Our next goal was to further establish the criterion validity of the GCES. Study 2 examines GCES-S scores and observer ratings of photographs in a sample known to be high in geek engagement: geek convention attendees.

## Study 2

In order to validate the GCES in the population for which it was intended, we gave the GCES-S as well as measures of narcissism and self-esteem to attendees at the 2013 meeting of Dragon*Con. If the GCES is a valid measure of geek engagement, we expect attendees at a major geek convention to score significantly higher on average than participants in non-geek specific populations (e.g., Samples A and B from MTurk). In addition, we wanted to further validate geek engagement as a construct by testing whether it was observable to naïve strangers. Studies have shown that outside observers can accurately perceive individual difference variables from photographs, [61] especially when the subject of the photograph has some control over the picture (e.g., pose, outfit, smile). If geek engagement is a valid construct, we can expect individuals higher in geek engagement to appear “geekier” than individuals lower in geek engagement in photos. Therefore, we took a photograph of each participant to examine whether observers’ perceptions of their appearance are consistent with their geek engagement score. Because narcissism can also be perceived through appearance [62], we also tested whether observers’ perceptions of their appearance are consistent with the hypothesized relationship between geek engagement and narcissism.

### Methods

#### Procedure

Participants (N = 202) were approached in downtown Atlanta, GA during the Dragon*Con geek convention. The researchers targeted persons wearing badges indicating they were attendees of the convention. Participants were informed as to the purpose of the study, gave consent by signing a consent form, and completed two pages of brief surveys. Then, with the participants’ consent, their picture was taken using a digital camera. Participants were given no specific instructions as to how to pose or whether to smile. They received no compensation for their participation.

#### Materials

The short form of the Geek Culture Engagement Scale (GCES-S; α = .79) created in Study 1 was used in Study 2. The GCES-S is a 14-item index of engagement in geek interests and activities. Participants indicated their engagement in each geek activity on a scale from 1 (*not at all)* to 5 (*very much)*. Due to a typographical error, about 50% of participants failed to rate the item Renaissance Fairs; however, this does not appear to have significantly affected the reliability of the full scale (α with Renfaires omitted = .79).

In order to reduce the burden of participation, we used a six item version of the Narcissistic Personality Inventory (NPI). Only the two highest loading questions on each factor of the Ackerman Split [53] for the NPI-13 [63] were used. The composite of these items showed an α of .73.

Again in the interest of time, the Single Item Self-Esteem Scale [64], consisting of the item “I see myself as someone who has high self-esteem,” was used. Participants endorsed this item on a Likert scale from 1 (*strongly disagree*) to 5 (*strongly agree*).

Photos of study participants were rated on three sets of criteria. All raters were members of our undergraduate research team at a large southeastern university. Each member of the team provided only one set of ratings, i.e., geek engagement raters were independent from subjective raters and the appearance rater.

*Appearance ratings* were obtained to examine whether individuals high in geek engagement differed appreciably in appearance, demeanor, or dress from those low in geek engagement. These ratings were assigned by a single rater and included ratings from Vazire et al. [62] as well as five additional items pertaining to costuming (i.e., “Does the person appear to be feminine (vs. masculine)?”; “Is the person wearing a costume?”; “Is the person wearing a t-shirt with a logo?; “Is the person striking a pose?”; and “Is the person smiling?”). These five items reflected either ways individuals could transmit their geek identity specifically (i.e., broadcasting their knowledge/devotion [8] to or conspicuous consumption of [1] a geek topic by wearing a costume or logo t-shirt) or basic appearance cues we felt were not adequately covered by the Vazire et al. ratings. The rater was instructed to focus on the person wearing the costume, rather than the character they were attempting to portray. The rater endorsed each item on a scale from 1 (*not at all*) to 5 (*very much*).

*Geek Engagement ratings* were obtained to see if trained observers can discern geek engagement and interests from outward appearance. These were assigned by five raters trained by the experimenter who received information on geek culture, fashion, and genres. Raters scored each picture on a scale from 1 (*not at all)* to 5 (*very much)* as to how much the participant appeared to hold each interest listed on the GCES-S, resulting in 20 scores for each picture. These scores were averaged to produce a Geek Engagement rating score. Intraclass correlations were above or close to .7 for LARP, Scifi, Anime, Manga, and Furry, and above .8 for Lolita. The rest were at or below .6.

*Subjective ratings* were obtained to examine the raters’ impressions of each participant’s personality and social status independent of their geek engagement. These were assigned by three raters who were given no instruction or background on geek culture. Participants endorsed ten items (i.e., “How narcissistic is this person?”; How geeky is this person overall?”; “How high is this person’s self-esteem?”; “How likeable is this person?”; “How self-centered is this person?”; “How attractive is this person?”, “How much social status does this person have?”; “How intelligent is this person?”; “How kind is this person?”; and “How caring is this person?”) on a Likert scale from 1 (*not at all)* to 5 (*very much*) for each picture. Intraclass correlations were above or close to .7 for Geeky, Self-Esteem, Attractive, and Social Status. The rest were at or below .6.

### Results

#### Average geek engagement

The mean GCES-S for conference attendees (M = 2.70, SD = .77) was significantly higher than both Sample A (M = 2.11, SD = .70), t(201) = 10.93, p < .001, *d* = .80, and Sample B (M = 1.76, SD = .76), t(201) = 17.36, p < .001, *d* = 1.22, from Study 1.

#### Zero-order correlations and regression

Zero-order correlations replicated findings from Study 1, with geek engagement related positively to narcissism, *r*(200) = .18, 95% CI [.04,.31], and unrelated to self-esteem, *r*(200) = -.17, 95% CI [-.30,.03]. In a multiple linear regression predicting geek engagement with gender, age, socioeconomic status, narcissism, and self-esteem, only age, narcissism, and self-esteem maintained significance. This further supports the findings of Study 1 in that age, narcissism, and self-esteem still predict geek engagement after controlling for gender or SES.

#### Appearance ratings

As seen in Tables 4 and 5, overall geek engagement was associated with wearing a costume, eyeglasses and makeup, and with putting a lot of preparation into ones appearance. Because these ratings included several items expected to differ by gender (e.g., femininity, makeup, cleavage), correlations were examined separately for both males and females, as well as for the full sample. Participants were classified as either male or female exclusively through self-report (i.e., they chose either the item “Male” or the item “Female” on our survey). Among women, higher geek engagement was associated with wearing a costume and makeup, striking a pose, being muscular and having put a lot of preparation into one’s appearance. Also among women, narcissism was associated with striking a pose and appearing feminine, while self-esteem was associated with appearing cheerful. Among men, higher geek engagement was negatively associated with wearing a t-shirt with a logo.

**Table 4:** Correlations between Appearance Ratings of Dragon*Con Photographs and Self-Esteem, Narcissism, and Geek Engagement.

	Feminine	Costume	Logo	Pose	Smiling
	Full Sample				
Self-Esteem	-.01 [-.15,.13]	-.06 [-.20,.08]	-.08 [-.22,.06]	.08 [-.06,.22]	**.14 [.01,.28]**
Narcissism	.02 [-.12,.15]	-.04 [-.17,.10]	-.07 [-.21,.07]	**.15 [.02,.29]**	.10 [-.04,.23]
Geek Engagement	.05 [-.08,.19]	**.16 [.02,.29]**	-.06 [-.19,.08]	.13 [-.01,.26]	-.03 [-.16,.11]
	Men				
Self-Esteem	.07 [-.13,.26]	.05 [-.14,.25]	-.13 [-.32,.07]	.19 [-.01,.37]	.08 [-.12,.27]
Narcissism	-.05 [-.24,.15]	-.13 [-.32,.07]	-.08 [-.27,.12]	.02 [-.18,.22]	.06 [-.14,.25]
Geek Engagement	.15 [-.05,.34]	.10 [-.09,.30]	**-.25 [-.43,-.06]**	.04 [-.16,.24]	-.04 [-.23,.16]
	Women				
Self-Esteem	.08 [-.12,.27]	-.11 [-.30,.09]	-.07 [-.27,.13]	-.07 [-.26,.13]	**.26 [.07,.44]**
Narcissism	.**20 [.01,.39]**	.07 [-.13,.27]	-.07 [-.27,.12]	**.24 [.05,.42]**	.18 [-.02,.37]
Geek Engagement	.13 [-.07,.32]	**.24 [.04,.42]**	.17 [-.03,.35]	**.23 [.04,.41]**	-.01 [-.21,.18]

Note: 95% Confidence intervals in brackets. Confidence intervals not containing 0 in bold.

**Table 5:** Correlations between Ratings of Dragon*Con photographs Based on Vazire et al. (2008) and Self-esteem, Narcissism, and Geek Engagement.

	Fashionable	Stylish	Expensive	Plain	Organized	Neat	Cheerful	Preparation	Makeup	Eyeglasses	Muscular	Skin
Full Sample												
SE	.09 [-.05,.23]	.07 [-.07,.20]	.04 [-.10,.18]	.11 [-.03,.24]	.01 [-.13,.14]	-.03 [-.17,.10]	.13 [.00,.27]	-.04 [-.17,.10]	.03 [-.10,.17]	.03 [-.10,.17]	.06 [-.07,.20]	.09 [-.05,.23]
N	.08 [-.06,.22]	.09 [-.05,.22]	.04 [-.10,.17]	-.03 [-.17,.10]	-.08 [-.22,.06]	-.01 [-.15,.13]	.08 [-.06,.21]	-.07 [-.21,.06]	-.01 [-.15,.13]	-.01 [-.15,.13]	.06 [-.07,.20]	.08 [-.06,.22]
GE	-.13 [-.26,.01]	-.08 [-.22,.06]	.07 [-.07,.20]	-.08 [-.22,.05]	-.13 [-.26,.01]	.11 [-.03,.24]	-.06 [-.19,.08]	**.17 [.03,.30]**	**.21 [.08,.34]**	**.21 [.08,.34]**	-.02 [-.15,.12]	-.13 [-.26,.01]
Men												
SE	.02 [-.18,.22]	.04 [-.15,.24]	.11 [-.09,.30]	.12 [-.08,.31]	.01 [-.19,.20]	-.12 [-.31,.08]	.05 [-.15,.24]	.02 [-.18,.21]	.07 [-.13,.26]	.05 [-.15,.24]	-.04 [-.24,.16]	.11 [-.09,.30]
N	.09 [-.11,.28]	.09 [-.11,.28]	.12 [-.08,.31]	.04 [-.15,.24]	-.09 [-.28,.11]	.00 [-.20,.19]	.00 [-.20,.20]	-.08 [-.27,.12]	-.02 [-.22,.17]	.01 [-.19,.20]	.10 [-.10,.29]	**.24 [.04,.42]**
GE	-.10 [-.29,.10]	-.10 [-.29,.10]	.16 [-.03,.35]	.01 [-.19,.20]	-.08 [-.27,.12]	.11 [-.09,.30]	-.11 [-.30,.09]	.11 [-.09,.30]	.16 [-.04,.34]	.05 [-.14,.25]	-.13 [-.32,.07]	-.15 [-.33,.05]
Women												
SE	.15 [-.05,.33]	.09 [-.11,.28]	.01 [-.19,.21]	.08 [-.11,.28]	-.01 [-.20,.19]	.05 [-.15,.25]	**.23 [.04,.41]**	-.03 [-.23,.17]	.02 [-.17,.22]	-.06 [-.26,.14]	-.09[-.28,.11]	.18 [-.02,.36]
N	.07 [-.13,.27]	.11 [-.09,.30]	.02 [-.18,.22]	-.11 [-.30,.09]	-.03 [-.23,.16]	-.06 [-.25,.14]	.17 [-.02,.36]	-.03 [-.23,.17]	.02 [-.18,.22]	-.03 [-.23,.17]	.00 [-.20,.20]	-.01 [-.20,.19]
GE	-.16 [-.35,.03]	-.07 [-.27,.13]	-.01 [-.21,.19]	-.18 [-.37,.01]	-.19 [-.37,.01]	.11 [-.09,.30]	-.02 [-.21,.18]	**.26 [.07,.44]**	**.27 [.08,.45]**	-.02 [-.22,.18]	**.24 [.05,.42]**	.13 [-.07,.32]

Note: 95% Confidence intervals in brackets. Confidence intervals not containing 0 in bold. SE = Self-esteem, N = Narcissism, and GE = Geek Engagement.

#### Geek engagement ratings

The average of observer ratings of the GCES-S items was negatively related to self-reports, *r*(200) = -.15, 95% CI [-.28,-.01]. Relationships between subscale scores and ratings can be found online at https://osf.io/u25x9/.

#### Subjective ratings

Judges’ ratings of narcissism, *r*(200) = .16, 95% CI [.02,.29], and self-centeredness, *r*(200) = .19, 95% CI [.05,.32], correlated positively with self-reports of narcissism, and ratings of self-esteem correlated positively with self-reported self-esteem, *r*(200) = .17, 95% CI [.03,.30], supporting the validity of these judges’ ratings. To the judges, individuals highest in geek engagement were seen as significantly more “geeky” as subjectively defined by the rater *r*(200) = .23, 95% CI [.10,.36].

### Discussion

Attendees at Dragon*Con scored significantly higher on geek engagement than our previous two samples, further supporting the validity of the GCES-S.

Although judges were accurately able to discern narcissism and self-esteem from appearance, they were unable to accurately assess geek engagement from photographs. These results imply that geek engagement as quantified by the GCES is not readily apparent from one’s physical appearance within the limited range of attendees at a geek convention. This may result from a possible difficulty in discerning differences among people of very high geek engagement, as a sample of individuals at a geek convention may present a restriction of range in geek engagement. However, a different set of judges were able to discern “geekiness” as they subjectively defined it which correlated positively with self-reported GCES scores. This highlights the utility of the GCES for quantifying what may only subjectively be discerned by trained observers.

Consistent with the great fantasy migration hypothesis, we again saw higher narcissism associated with higher self-reported geek engagement. However, although participants with high geek engagement appeared to have put more preparation in their appearance and women high in geek engagement wore more makeup (both of which can be signs of narcissism [62]), these ratings were not themselves associated with self-reported narcissism, suggesting that these ratings (along with the rest of the Vazire et al. ratings) may not be valid cues of narcissism in geek populations.

In Studies 1 and 2 we have established that self-reported engagement in geek activities relates consistently to several individual differences variables. The relationship between geek engagement and narcissism is consistent with the great fantasy migration hypothesis; however, the core of this hypothesis is that individuals high in narcissism are escaping unsatisfactory engagement in real life by engaging in fantasy themed activities. In Study 3 we explore whether engagment in geek culture is associated with less engagement in civic activities and planning for the future. We predict a tradeoff in that the more time and resources an individual has devoted to geek activities (i.e., the more migrated the individual is), the less time and resources he or she will have available to engage in civic behavior or show concern about his or her future. We thus predicted a negative relationship between geek engagement and civic engagement as well as future orientation.

## Study 3

In Studies 1 and 2, engagement in geek culture activities related positively to narcissism. These results provide preliminary support for the great fantasy migration hypothesis, which predicts that persons high in narcissism may migrate to the fantasy worlds provided in geek culture, and thus become less engaged in real life activities. We further examined this hypothesis by measuring geek culture engagement, life goals, and civic engagement in a sample of normal adults to test whether geek cultural engagement would be associated with less civic engagement and lower interest in life goals pertaining to career, family, and political achievement. We also tested whether geek engagement was associated with lower future orientation—we predicted that individuals showing less engagement with real life would also show less concern for the future and potential consequences of their actions.

### Methods

#### Procedure

Again, participants (N = 348) indicated their consent by clicking “I consent” on a consent script and completed the measures via an online survey hosting website before being compensated via MTurk. The same generic posting was used as in Study 1 Sample B.

#### Materials

We used the GCES (α = .93) and NPI (α = .89) described in previous studies.

#### Life Goals

Participants endorsed 34 items (see Table 6) from Monitoring the Future [65] as used in Twenge, Campbell, and Freeman [66] on a scale from 1 (*not important*) to 4 (*extremely important*). Items included a range of potential life goals such as family (e.g., “Having a good marriage and family life”), activist (e.g., “Participating in a community action program”), financial (e.g., “Having lots of money”), and recreational (e.g., “Having plenty of time for recreation and hobbies”) goals.

**Table 6:** Correlations between Geek Engagement and Life Goals Items from Twenge, Campbell, & Freeman (2012) and Civic Engagement Scores.

	Geek Engagement		Geek Engagement		Geek Engagement
1. Finding purpose and meaning in my life.	-.04 [-.14,.07]	1. Being very well off financially.	.08 [-.03,.18]	15. Becoming accomplished in one of the performing arts (i.e. acting, dancing)	**.40 [.31,.48]**
2. Being a leader in my community.	**.25 [.14,.34]**	2. Developing a meaningful philosophy of life.	.08 [-.03,.18]	16. Influencing the political structure.	**.33 [.24,.43]**
3. Being close to parents and relatives.	**-.13 [-.23,-.03]**	3. Keeping up to date with political affairs.	**.12 [.02,.22]**	17. Becoming successful in a business of my own.	**.22 [.11,.31]**
4. Being able to find steady work.	-.05 [-.15,.06]	4. Having administrative responsibility for the work of others.	**.27 [.17,.36]**	18. Helping others who are in difficulty.	.07 [-.04,.17]
5. Having strong friendships.	.01 [-.10,.11]	5. Becoming involved in programs to clean up the environment.	**.29 [.19,.38]**	19. Writing original works (i.e. poems, novels, short stories)	**.37 [.27,.46]**
6. Having a good marriage and family life.	**-.14 [-.24,-.03]**	6. Becoming a community leader.	**.25 [.14,.34]**	20. Creating Artistic Work	**.27 [.17,.37]**
7. Having lots of money.	**.12 [.01,.22]**	7. Raising a family.	-.07 [-.17,.04]	Media Awareness	.07 [-.04,.17]
8. Working to correct social and economic inequalities.	**.18 [.07,.28]**	8. Obtaining recognition from my colleagues for my contributions to my special field.	**.18 [.08,.28]**	Political Behavior	**-.27 [-.37,-.17]**
9. Discovering new ways of experiencing things.	**.22 [.12,.32]**	9. Participating in an organization like the Peace Corps or Americorps/VISTA.	**.37 [.28,.46]**	Political Trust	**.33 [.24,.42]**
10. Being able to give my children better opportunities than I've had.	-.06 [-.17,.04]	10. Influencing social values.	**.23 [.13,.33]**	Social Trust	-.05 [-.15,.06]
11. Being successful in my line of work.	.08 [-.02,.18]	11. Becoming an authority in my field.	**.25 [.14,.34]**	Civic Organizations	**.20 [.10,.30]**
12. Having plenty of time for recreation and hobbies.	.01 [-.10,.11]	12. Making a theoretical contribution to science.	**.36 [.27,.45]**	Civic Knowledge	**-.12 [-.22,-.01]**
13. Making a contribution to society.	.03 [-.07,.14]	13. Participating in a community action program.	**.25 [.15,.35]**		
14. Getting away from this area of the country.	**.26 [.16,.35]**	14. Helping to promote racial understanding.	**.25 [.15,.34]**		

Note: 95% Confidence intervals in brackets. Confidence intervals not containing 0 in bold.

#### Civic engagement

The 25 items used by Jennings and Zeitner [67] formed our measure of civic engagement. Participants reported their media usage on a scale from 1 (*not at all*) to 4 (*almost daily*), indicated their involvement in political campaigns and civic organizations (e.g., church, labor unions, political groups, sports teams) via yes/no questions, demonstrated political knowledge through a brief test, and answered several multiple choice questions concerning their trust in others and in the government.

#### Future orientation

The Future Orientation Scale (FOS) [68] is a 15-item measure of the tendency to attend to and plan for future consequences. Participants were given 15 pairs of opposing statements (e.g. “Some people like to plan things out one step at a time,” “Other people like to jump right into things without planning them beforehand”) and both chose a statement and rated that statement as either “*really true for me*” or “*sort of true for me*.” In addition to the full scale score (α = .83), the FOS has three subscale scores: Planning Ahead (α = .71), Time Perspective (α = .60), and Anticipation of Future Consequences (α = .70).

### Results

#### Narcissism

As with the previous studies, narcissism was again correlated positively with geek engagement, *r*(347) = .30, 95% CI [.19,.41]. In addition, narcissism was marginally positively related to media awareness, *r*(347) = .11, 95% CI [.00,.22], positively related to political trust, *r*(347) = .20, 95% CI [.09,.31], and engagement in civic organizations, *r*(347) = .13, 95% CI [.02,.24], but negatively correlated with political behavior, *r*(347) = -.16, 95% CI [-.27,-.05] and civic knowledge, *r*(347) = -.16, 95% CI [-.27, -.05]. Narcissism was unrelated to future orientation, *r*(347) = -.08, 95% CI [-.19, .03].

#### Life goals

The link between geek culture engagement and life goals was mixed. As seen in Table 6, individuals high in geek engagement endorsed items reflecting a desire for power or status (e.g., “Having administrative responsibility over the work of others,” “Becoming an authority in my field,” “Influencing the political structure”), career advancement (e.g., “Becoming successful in a business of my own”), activism or humanitarian concerns (e.g., “Helping to promote racial understanding,” “Working to correct social and economic inequalities”), sensation seeking (e.g., “Discovering new ways of experiencing things,” “Getting away from this area of the country”) and artistic pursuits (e.g., “Becoming accomplished in one of the performing arts”). However, in a series of multiple regressions predicting each life goal with geek engagement, future orientation, narcissism, age, gender, and SES, geek engagement no longer predicted the life goals “Being a leader in my community,” “Having lots of money,” “Working to correct social and economic inequalities,” “Keeping up to date with political affairs,” “Having administrative responsibility over the work of others.” “Becoming a community leader,” “Obtaining recognition from my colleagues for my contributions to my special field,” and “Becoming an authority in my field.” The majority of these items (i.e., items pertaining to leadership, status, and recognition) were most likely related to geek engagement through narcissism.

Items reflecting family goals were either unrelated (e.g., “Raising a family,” “Having a good marriage and family life”) or negatively related (e.g., “Being close to parents and relatives”) to geek engagement. Geek engagement was also unrelated to the desire for meaning (e.g., “Developing a meaningful philosophy of life,” “Finding purpose and meaning in my life”).

#### Civic engagement

As seen in Table 6, individuals high in geek engagement appear to participate in civic organizations and have high trust in the government, but show less political knowledge and are markedly less involved in the political process (campaigns, voting, etc.). In a series of multiple regressions predicting each index with age, gender, SES, geek engagement, narcissism, and future orientation, geek engagement no longer predicted political knowledge. These results indicate some disengagement from political behavior but not from other forms of civic behavior, such as special interest groups and activism. Individuals higher in geek engagement were significantly more likely to hold membership in neighborhood associations, *r*[347] = .16, 95% CI [.05,.26], nonpartisan groups, *r*[347] = .13, 95% CI [.03,.23], ethnic, racial, or nationality associations, *r*[347] = .22, 95% CI [.11,.31], support or self-help groups, *r*[347] = .13, 95% CI [.03,.23], and music or art groups *r*[347] = .23, 95% CI [.13,.33].

#### Future orientation

Geek engagement did not show a significant association with future orientation, *r*(347) = -.07, 95% CI [-.17,.05], nor with its subscales Planning Ahead, *r*[347] = -.10, 95% CI [-.20,.01], Time Perspective, *r*[347] = .03, 95% CI [-.08,.13], and Anticipation of Consequences, *r*[347] = -.07, 95% CI [-.17,.04].

### Discussion

Overall, the results of this study are inconsistent with the civic engagement aspects of the great fantasy migration hypothesis. There was a positive association with narcissism. Likewise, geek engagement was associated with disengagement from political behavior and family oriented goals. However, high geek engagement scores are still positively associated with many other life goals and participation in non-political civic organizations, suggesting higher civic engagement in most areas for geeks. Although many of the civic groups reported (e.g., self-help, ethnic and nationality organizations) appear unlikely to be geek related, it is possible that geek organizations, such as volunteering at conventions or geeky art or musical groups, provide more opportunities for geeks to be engaged. In addition, geek engagement was unrelated to future orientation. This may be because geeks are equally future oriented toward their geek behavior as non-geeks are to real life, or it may reflect normal engagement with real life. Together these results suggest that although narcissism continues to reliably predict geek engagement, the aspects of the great fantasy migration that predict reduced engagement in real life need to be revised.

Studies 1 through 3 focused on the great fantasy migration hypothesis. Although we found some supporting evidence for this hypothesis, geek behavior may be influenced by other processes as well. Studies 4 and 5 will examine the belongingness hypothesis. We predict that individuals engage in geek culture because they believe it will fulfill their needs for belongingness. In Study 4, we examine belongingness needs as a motivation to both engage in geek behavior and to identify with geek culture. We predicted that individuals who anticipated acceptance from others when engaging in geek behaviors would be more likely to identify as a geek and to engage in geek behavior than individuals who did not. We thus predicted a positive relationship between geek engagement and geek identity.

## Study 4

The goals of Study 4 are twofold. First, we tested the belongingness hypothesis through an adaptation of Leary et al.’s [69] belongingness paradigm. Participants rated each activity from the GCES in terms of how they would feel when they participated in the activity as well as how they believed others would react to them. Based on Sociometer Theory [69], participants will report feeling higher self-esteem and more positive feelings when performing activities that they believe would lead others to accept them. If the belongingness hypothesis holds true, higher geek involvement will be associated with believing others who are important to them would accept them for engaging in geek activities and with feeling more positively when engaging in the same activities.

Second, we sought to further validate the GCES by measuring its relationship to reported geek behaviors (e.g., LARPs and conventions attended, role-playing games played) and to geek identity. To accomplish the latter, we developed the Geek Identity Scale (GIS), a brief measure of the extent to which one identifies as a geek and as part of geek culture. We predicted that higher scores on the GCES would be associated with higher frequency of geek behaviors and higher scores on the GIS.

### Method

#### Participants and procedure

Participants (N = 334) indicated their consent by clicking “I consent” on a consent script and completed the measures via an online survey hosting website before being compensated via MTurk.The same generic posting was used as in Study 1, Sample B.

#### Materials

The full 37-item GCES was used for this study. The scale showed a Cronbach’s α of .91.

To measure geek behaviors, we asked participants to quantify how often they engaged in each of the activities or lifestyles listed. Participants responded to 39 questions with how often they engaged in each activity in either the last day, week, month, or year, depending on the activity. Time frame was contingent on the availability of the behavior (e.g., one can spend several hours on internet forums a day but can only attend so many conventions in a year).

Participants responded to the Geek Identity Scale (GIS)—ten questions measuring whether they identified as a geek and saw participation in geek culture as part of their identity. The full GIS can be found in the appendix. Questions were on a Likert scale (1 = *Strongly Disagree*; 5 = *Strongly Agree*) and showed a Cronbach’s α of .97.

Consistent with Leary et al. [69], participants rated the geek behaviors mentioned above five times: first, how they felt “the people important in their life” would react to them if they engaged in that activity (1 = *many other people would reject or avoid me*; 5 = *many other people would accept or include me*) and then how they imagine they would feel if they engaged in the activity on four dimensions (bad/good, ashamed/proud, worthless/valuable, and dejected/happy). Each dimension was rated on a 5-point scale with 1 indicating the negative emotion and 5 indicating the positive.

### Results

#### Geek activities and identity

To assess whether Geek Engagement was associated with self-identification as a geek and geek behavior, we correlated each GCES activity with the self-reported frequency of performing that activity, as well as with the summed GIS scores. Consistent with our predictions, each activity or lifestyle listed in the GCES positively predicted the frequency of engaging in that activity or lifestyle (see Table 7) and full scale GCES score significantly predicted the frequency of engaging in each activity or lifestyle (*r*’s = .11-.45). In addition, full scale GIS score positively predicted geek engagement, *r*(333) = .47, 95% CI [.38, .55], confirming that those high in geek engagement are more likely to explicitly identify as a geek. Finally, we split the sample into specialists who reported high engagement in only one or two geek activities (N = 128) and generalists who engaged in three or more geek activities (N = 141). Specialists (*M =* 2.55) scored significantly lower in geek identity than generalists (*M* = 3.32), *t*(140) = 8.52, p < .001, further supporting our conception of geek identity as being higher when individuals engage in more geek activities.

**Table 7:** Correlations between Geek Engagement and Frequency of Geek Activities.

	Geek Engagement		Geek Engagement		Geek Engagement
LARP events per mo.	**.45 [.36,.53]**	Hours spent participating in theater per year	.11 [.00,.22]	Hours spent Filking per mo.	**.21 [.10,.31]**
Table Top Role Playing Game sessions per mo.	**.42 [.33,.50]**	Hours spent on Creative Writing per mo.	**.14 [.03,.24]**	Hours spent watching Cinema per mo.	**.35 [.25,.44]**
Hours spent Computer/Console Gaming per mo.	**.25 [.15,.35]**	Hours spent on Social Networking websites per week	**.22 [.11,.32]**	Hours spent watching Joss Whedon series per mo	**.37 [.27,.46]**
Hours spent on Cosplay per mo.	**.36 [.26,.45]**	Hours spent on Fantasy-themed activities per mo.	**.35 [.26,.44]**	Showings of the Rocky Horror Picture Show participated in per year	**.35 [.25,.44]**
Hours spent posting in internet forums per week	**.18 [.08,.28]**	Hours spent on Sci-Fi themed activities per mo.	**.36 [.27,.45]**	Meetings of Skeptic societies attended per year	**.41 [.32,.49]**
Conventions attended per year	**.42 [.33,.50]**	Hours spent watching Anime per mo.	**.21 [.11,.31]**	Hours spent participating in the Lolita lifestyle per mo.	**.24 [.13,.34]**
Renaissance fairs attended per year	**.44 [.35,.53]**	Hours spent reading Manga per mo.	**.21 [.10,.31]**	Hours spent participating in the Goth/Punk Rock lifestyle per mo.	**.30 [.20,.39]**
SCA and other historical reenactment events attended per year	**.37 [.27,.46]**	Hours spent reading or trading Comic Books per mo.	**.36 [.26,.45]**	Hours spent participating in the Furry (anthro, etc.) lifestyle per mo.	**.29 [.19,.39]**
Hours spent on weapons collecting per mo.	**.16 [.05,.26]**	Hours spent on Horror themed activities per mo.	**.17 [.06,.27]**	Hours spent participating in the Pagan (i.e., Wiccan, Norse, etc.) religion per mo.	**.22 [.12,.32]**
Hours devoted to paranormal Investigation per mo.	**.26 [.16,.36]**	Hours spent watching, listening to or acting in Broadway Musicals per mo.	**.24 [.13,.33]**	Hours spent practicing BDSM per mo.	**.38 [.28,.47]**
Hours spent on puppetry per mo.	**.29 [.19,.38]**	Hours spent on Alternative History-themed activities per mo.	**.37 [.27,.46]**	How many BDSM/kink events attended per year	**.40 [.31,.49]**
Hours spent on robotics per mo.	**.12 [.02,.23]**	Hours spent watching non-Anime Animation per mo.	**.19 [.09,.29]**	Polyamorous (consentually nonmonogamous) relationships	**.28 [.18,.38]**
Hours spent learning about robotics per mo.	**.21 [.10,.31]**	Hours spent watching British Series per mo.	.11 [.00,.22]	Polyamorous relationship partners	**.21 [.10,.31]**

Note: 95% Confidence intervals in brackets. Confidence intervals not containing 0 in bold.

#### Belongingness

To assess whether engagement in geek activities was associated with belongingness motives, we correlated the reject me/accept me rating for each item on the GCES with the feelings ratings for that activity (e.g., the reject me/accept me rating for Comic Books was correlated with the bad/good, ashamed/proud, worthless/valuable, and dejected/happy ratings for Comic Books). This was done instead of the within-person correlation analysis performed in Leary et al. [69] in order to simplify the analyses. For all geek behaviors listed, the extent to which participants expected those important in their lives to accept vs. reject them for performing each activity positively predicted whether they would feel good vs. bad (*rs* = .36-.65), proud vs. ashamed (*rs* = .25-.61), valuable vs. worthless (*r*s = .27-.61) and happy vs. dejected (*r*s = .30-.67). We also correlated each GCES activity with the corresponding reject me/accept me rating to see whether engagement in that activity was associated with expected acceptance or rejection. The degree to which participants expected those important in their lives to accept them for performing each activity positively predicted the frequency of their engaging in that activity (*r*s = .11-.36) with the exception of LARPing, theater, use of social networking sites, BDSM, and participation in live casts of the *Rocky Horror Picture Show*. Finally, we computed an average of the accept me/reject me ratings as an index of anticipated acceptance of others when engaging in geek activities. This average was positively related to geek identity, *r*(333) = .26, 95% CI [.16, .36], suggesting that belongingness motives are associated with having a stronger geek identity.

### Discussion

People appear to have positive self-feelings when engaging in geek activities to the extent that they expect important people in their life to accept them for doing so. In addition, people appear to identify more strongly as a geek when they expect others to accept them for engaging in geek activities. Although these data cannot establish causation, they are not inconsistent with the hypothesis that people engage in geek activities and identify with geek culture at least in part because of belongingness motives.

In addition, the GCES significantly predicts greater reported frequency of geek behaviors as well as greater identification as a geek. These findings support the criterion validity of the GCES in that GCES scores predict actual behavior and self-identification as a geek. In addition, the relationship between geek engagement and geek identity imply that geek culture includes a source of personal identity, consistent with Social Identity Theory [26] and that identification as a geek increases as engagement in more geek activities increases. Future research should examine the implications of this identity, including but not limited to whether geek identity implies identifying as an outsider with reference to mainstream culture and whether there is stigma associated with this identity. However, such hypotheses are beyond the scope of this paper.

Study 4 provides support for the belongingness hypothesis by showing engagement in geek culture is positively related to the belief that others will accept them for doing so. Study 5 tests the prediction that individuals who engage in geek culture will have stronger friendship ties with individuals who are similarly engaged, especially with regard to specific geek interests, than with those who do not share geek interests.

## Study 5

The belongingness hypothesis proposes that individuals engaging in geek activities form ties with others through those activities. Based on this hypothesis, we would expect to see geeks naming predominately other geeks as members of their social networks, and for their strongest ties to be with others who share their same specific geek interests (e.g., hobbies with hobbies, lifestyles with lifestyles). In Study 5, we conducted a social network analysis of the egocentric networks of a sample of normal adults on MTurk. Participants nominated up to 30 of the people closest to them and rated those persons on each of the subscales of the GCES. We predicted strong homophily among geeks—that geeks’ social networks would consist primarily of others with similar geek engagement scores, and that among high scoring geeks their ties would be closest with those who shared similar obscure interests.

### Methods

#### Procedure

The same posting used to target geeks in Study 1, Sample A was used to recruit participants on Amazon MTurk. Participants (N = 181) indicated their consent by clicking “I consent” on a consent script and completed the measures via an online survey hosting website before being compensated via MTurk.

#### Materials

In addition to the GCES (α = .91) participants reported egocentric network data, with each participant (ego) instructed to report 30 friends and family who are important in their lives (alters). The number 30 was chosen because it has been shown to be the optimal number of alters needed to accurately discern the nature of the network [70] However, 45.7% of the sample did not comply with this instruction and listed less than 30 alters. These participants listed an average of 9.23 alters each. Participants also rated each alter’s engagement in each subscale of the GCES on a scale from 1 (*not at all*) to 5 (*very much*). Finally, participants answered questions about the relationships among their alters. Each alter pairing was rated on a scale ranging from 1 (*strangers*) to 3 (*very close*).

### Results

#### Social network parameters: Density, degree centrality

Density and degree centrality scores were calculated for each ego’s network using UCINET software [51]. The ego’s geek culture engagement scores were then correlated to these measures. Density reflects the proportion of the number and strength of connections in a network to the total number and strength of connections possible. The higher the density score, the more strong connections are present. Geek engagement was not significantly correlated to density, showing that individuals engaged in geek culture were not more likely to have interrelated networks where everyone knows everyone else. Degree centrality reflects the degree to which any one or few alters are central, or a connecting point, for many other alters in the network. Geek engagement was not related to degree centrality, showing that those high in geek engagement were not more likely to be central in their social networks.

#### Homophily analyses

Homophily between each ego and his or her alters was calculated by correlating the ego’s geek engagement score with the average geek engagement score of his or her network. As seen in Table 8, significant zero-order correlations suggested a strong homophily between all geeks regardless of specific interests. Because the facets of the GCES (see Study 1) are strongly correlated with one another, we also ran a series of regressions controlling for shared variance between the subscales. Table 9 shows the same relationships as Table 8 when controlling for shared variance between subscales. These data show a clear pattern of homophily between geeks who share the same interests in all subscales of the GCES with the exception of hobbies. In addition, strong homophily remained between individuals who share interests that may be compatible, (e.g., roleplaying and theater).

**Table 8:** Correlations between Ego Geek Scores and the Average Geek Scores of their Networks.

Ego Scores	Lifestyles Avg	Genres Avg	Hobbies Avg	Japanese Avg	Theater Avg	Role Playing Avg	Puppetry Robotics Avg	Geek Engagement Avg
Lifestyles	**.53 [.42,.63]**	**.38 [.25,.50]**	**.27 [.12,.40]**	**.42 [.29,.53]**	**.38 [.24,.49]**	**.34 [.20,.46]**	**.36 [.23,.48]**	**.51 [.39,.61]**
Genres	.10 [-.05,.24]	**.38 [.24,.49]**	**.33 [.19,.45]**	**.18 [.03,.32]**	**.19 [.05,.33]**	**.06 [-.09,.20]**	.04 [-.11,.18]	**.24 [.10,.37]**
Hobbies	**.45 [.33,.56]**	**.39 [.26,.51]**	**.32 [.18,.44]**	**.41 [.28,.52]**	**.38 [.25,.50]**	**.41 [.28,.52]**	**.36 [.23,.49]**	**.53 [.41,.62]**
Japanese	**.41 [.28,.52]**	**.43 [.31,.55]**	**.20 [.06,.34]**	**.56 [.45,.65]**	**.30 [.16,.42]**	**.31 [.17,.44]**	**.26 [.12,.39]**	**.47 [.35,.58]**
Theater	**.30 [.17,.43]**	**.37 [.23,.49]**	**.34 [.20,.46]**	**.28 [.14,.41]**	**.47 [.35,.57]**	**.20 [.06,.34]**	**.25 [.10,.38]**	**.42 [.29,.53]**
Role Playing	**.45 [.32,.56]**	**.29 [.16,.42]**	**.29 [.15,.42]**	**.30 [.17,.43]**	**.36 [.22,.48]**	**.54 [.42,.63]**	**.33 [.20,.46]**	**.48 [.36,.58]**
Puppetry Robotics	**.36 [.23,.48]**	**.30 [.16,.42]**	**.16 [.01,.30]**	**.36 [.22,.48]**	**.40 [.27,.52]**	**.34 [.20,.46]**	**.42 [.29,.53]**	**.42 [.30,.54]**
Geek Engagement	**.50 [.38,.60]**	**.50 [.38,.60]**	**.39 [.26,.51]**	**.47 [.35,.58]**	**.48 [.36,.58]**	**.39 [.26,.51]**	**.37 [.24,.49]**	**.59 [.49,.68]**

Note: 95% Confidence intervals in brackets. Confidence intervals containing 0 in bold.

**Table 9:** Beta Coefficients between Ego Geek Scores and the Average Geek Scores of their Networks Controlling for Correlation Between Subscales.

Ego Scores	Lifestyles Avg	Genres Avg	Hobbies Avg	Japanese Avg	Theater Avg	Role Playing Avg	Puppetry Robotics Avg
Lifestyles	**.37 [.24,.49]**	**.18 [.03,.32]**	.11 [-.04,.25]	**.16 [.01,.30]**	.09 [-.06,.23]	.09 [-.06,.23]	.13 [-.02,.27]
Genres	-.06 [-.20,.09]	**.24 [.09,.37]**	**.23 [.09,.37]**	-.01 [-.16,.14]	-.04 [-.19,.10]	-.06 [-.21,.08]	-.09 [-.23,.06]
Hobbies	-.07 [-.21,.08]	-.01 [-.15,.14]	.06 [-.09,.20]	.02 [-.12,.17]	-.14 [-.28,.01]	.01 [-.14,.16]	.03 [-.11,.18]
Japanese	**.19 [.05,.33]**	**22 [.08,.36]**.	-.02 [-.16,.13]	**.44 [.32,.55]**	.07 [-.07,.22]	.13 [-.02,.27]	.05 [-.09,.20]
Theater	-.01 [-.15,.14]	04 [-.11,.18]	10 [-.05,.24]	-.03 [-.17,.12]	**.35 [.22,.48]**	-.09 [-.23,.06]	.02 [-.13,.17]
Role Playing	.**26 [.11,.39]**	.06 [-.08,.21]	**.16 [.02,.30]**	.04 [-.11,.18]	**.16 [.01,.30]**	**.46 [.34,.57]**	.12 [-.03,.26]
Puppetry Robotics	.03 [-.12,.17]	.03 [-.11,.18]	-.08 [-.22,.07]	.07 [-.07,.22]	**.19 [.04,.32]**	.07 [-.08,.21]	**.26 [.12,.39]**

Note: 95% Confidence intervals in brackets. Confidence intervals not containing 0 in bold.

### Discussion

The results of Study 5 appear to support the belongingness hypothesis. Geeks appear to form the strongest ties with those sharing similar specific geek interests, but also are more likely to form strong ties with other geeks who have similar interests. Although these results should be interpreted with caution due to noncompliance issues (i.e., the majority of participants not listing the requested number of alters), this general geek homophily is consistent with past research conceptualizing geek culture as using geek interests as social currency [8]. Future research should examine whether this homophily results from shared norms, beliefs, and values between the different fandoms in geek culture.

Studies 1 through 5 focused on the great fantasy migration and belongingness hypotheses. In Studies 6 and 7, we turned our attention to our third hypothesis, the need for engagement hypothesis, which proposes that individuals high in need for stimulation and creative outlets (i.e., individuals high in need for cognition, intelligence, openness, creativity, and sensation seeking) would be more likely to engage in geek culture.

## Study 6

In Study 6 we measured intelligence, fantasy proneness, and several known predictors of creativity (e.g., schizotypal personality and dissociative traits) along with geek culture engagement in a general sample of adults.

Positive associations between the immersive aspects of geek culture engagement and fantasy proneness would be consistent with the great fantasy migration hypothesis. We predicted that individuals who have functional levels of fantasy proneness would be more likely to engage in geek culture because of its fantasy-themed content in geek activities. Positive associations between geek culture engagement and fantasy proneness, crystallized and fluid intelligence, need for cognition, and sensation seeking would be consistent with the need for engagement hypothesis. Because many fan groups distinguish themselves as having more active engagement with their media (e.g., [10]), we predicted geeks would be individuals who had higher need for intellectual (e.g., need for cognition) and emotional (e.g., sensation seeking) stimulation.

We also included the NPI and Five Factor Model Checklist.

### Methods

#### Participants and procedure

Again, participants (N = 226) indicated their consent by clicking “I consent” on a consent script and completed the measures via an online survey hosting website before being compensated via MTurk. The same generic posting was used as in Study 1, Sample B.

#### Materials

In addition to the GCES (α = .94), NPI (α = .91) and Five Factor model checklist (N [α = .77] E [α = .70] O [α = .68] A [α = .68] C [α = .81]) we included the following measures.

The Creative Experiences Questionnaire (CEQ) [71] is a 25-item measure of fantasy prone personality (α = .85). Participants answered a series of yes/no questions (e.g., “I prefer watching educational to entertainment programs”) about their fantasies, magical beliefs, and childhood experiences. The total number of “yes” answers indicated their level of fantasy proneness.

The Dissociative Experiences Questionnaire (DEQ) [72] is a 27-item measure of dissociative symptomology (α = .95). Participants indicated through a sliding scale what percentage of the time they experience each item (e.g., “Some people have the experience of looking in a mirror and not recognizing themselves”) in their daily lives.

The Schizotypal Questionnaire (STQ) [73] is a 37-item measure of schizotypal personality (α = .92). Participants answered yes/no questions such as “Do you believe in telepathy?” and “Do you feel it is safer to trust nobody?” The total number of “yes” answers indicated their level of schizotypal symptomology.

General intelligence has been shown to be made up of two main factors, crystallized intelligence (or accumulated knowledge) and fluid intelligence (ability to work with information). Intelligence is best assessed by measuring both factors [74]. The Shipley Institute of Living Scale [75] is a brief self-administered measure of both crystallized and fluid intelligence. The crystallized subscale consists of 40 vocabulary terms of increasing difficulty. Participants chose a synonym from four answer choices for each term. The fluid subscale consists of 20 pattern recognition items. Participants discerned the relationship between the numbers, letters, or words in each item and provided the next item in the series. For both subscales, we used the number of correct answers as their intelligence score.

The Need for Cognition scale (NFC) [31] is a 34-item measure (α = .95) of need for cognition, or the tendency to take pleasure in thinking for its own sake. Participants rated statements such as “I really enjoy a task that involves coming up with new solutions to problems” on a Likert-type scale from -4 (*very strong disagreement*) to +4 (*very strong agreement*). Because of computer error, item 5 was omitted from the NFC scale. However, it appears to show excellent reliability.

The Sensation Seeking Scale (SSS) [76] consists of 34 forced-choice items (α = .82). Participants chose between statements such as “I prefer friends who are excitingly unpredictable” versus “I prefer friends who are reliable and predictable.” The number of sensation seeking choices made comprised the participants’ total score.

### Results

We assessed relationships between all variables using zero-order correlations and linear regressions. Geek engagement continued to be positively related to narcissism, *r*(226) = .24, 95% CI [.11,.36], neuroticism, *r*(226) = .23, 95% CI [.10, .35], and openness to experience, *r*(226) = .28, 95% CI [.16,.40]. Consistent with the great fantasy migration and desire for engagement hypotheses, geek engagement was also positively related to fantasy proneness, *r*(226) = .49, 95% CI [.39,.58], dissociative symptoms, *r*(226) = .59, 95% CI [.49,.67], and schizotypal personality, *r*(226) = .40, 95% CI [.29,.51]. Likewise, geek engagement was positively associated with sensation seeking, *r*(226) = .32, 95% [.20,.43], supporting the assertion that individuals more in need of stimulation tend to engage more strongly in geek culture. In contrast, geek engagement was negatively related to fluid intelligence, *r*(226) = -.17, 95% CI [-.30,-.04], and crystallized intelligence, *r*(226) = -.29, 95% CI [-.40,-.16], and unrelated to need for cognition.

Due to high intercorrelation between the constructs in this study (*r*s = .25-.70), we tested the relationship between geek engagement and intelligence, need for cognition, sensation seeking, schizotypal personality and dissociation in separate regressions along with age, gender, SES, and the Big Five personality traits. Sensation seeking and fluid intelligence ceased to be significant predictors of geek engagement when controlling for these variables.

Thus overall support for the desire for engagement hypothesis was mixed–there was high openness but lower intellectual ability in this sample.

### Discussion

The results of Study 6 suggest that those individuals most engaged in geek culture are more likely to report traits associated with narcissism, openness, neuroticism, and fantasy proneness, and have tendencies toward dissociation and schizotypal personality, but have lower crystallized intelligence than individuals lower in geek engagement. This pattern is consistent with the great fantasy migration hypothesis, but only partially consistent with the desire for engagement hypothesis, as geek engagement showed strong relationships with those constructs related to creativity (i.e., fantasy proneness, openness, dissociation and schizotypy) but not those related to intellectual and emotional stimulation (i.e., need for cognition and sensation seeking) when controlling for demographics and personality. This suggests those engaged in geek culture only need engagement in terms of creative outlets, rather than stimulation. In addition, the negative relationship with crystallized intelligence, although persistent when controlling for demographic variables, needs more research to be fully understood. The Shipley uses a vocabulary test as a proxy for crystallized intelligence. This may reflect reduced education or reduced verbal ability rather than reduced cognitive ability. More research with a more in depth intelligence scale is warranted.

In addition to the traits measured in Study 6, the need for engagement hypothesis predicts that creative individuals may be more likely to engage in geek culture. Study 7 examines this prediction.

## Study 7

Another individual difference variable associated with need for engagement is creativity. Creative people are often said to require stimulation and novelty [34], and in addition to having stimulating and novel themes (e.g., fantasy, science fiction) geek culture activities (such as constructing costumes, writing storylines for role playing games, and portraying popular characters through cosplay) offer a plethora of creative outlets. In Study 7, we measured geek culture engagement along with several aspects of creativity including values and attitudes toward creativity, creative activities and behaviors, and the generation of ideas. Because we had not yet measured education in regards to geek engagement, we also included education in our demographics for this study. A positive relationship between geek engagement and creativity would be consistent with the desire for engagement hypothesis.

### Methods

#### Procedure

Again, participants (N = 396) indicated their consent by clicking “I consent” on a consent script and completed the measures via an online survey hosting website before being compensated via MTurk. The same generic posting was used as in Study 1, Sample B.

#### Materials

In addition to the GCES (α = .91) and the GIS (α = .96), individuals were asked to indicate their level of education on a scale from 1 (*less than a high school diploma*) to 5 (*Ph*.*D*. *and beyond)* and three scales from the Runco Creativity Assessment Battery (rCAB) [77] were used to assess behavioral, ideational, and attitudinal aspects of creativity. These scales are as follows:

The Creative Activity Checklist is a 65-item list (α = .98) of creative activities and accomplishments (i.e., “Remixed music on a computer,” “Designed a website”). Participants indicated whether they have engaged in each activity (a) never (b) *once* in school or work as an assignment (c) *more than once* in school or work (d) *once* on their own or (e) *more than once* on their own. Participants receive two scores: school or work (α = .97) and on their own (α = .97).

The Runco Ideational Behavior Scale Short Form (RIBS-S; α = .83) is a 19-item scale designed to measure the frequency with which one generates original ideas. Participants rated how often items such as “I hear songs and think of different or better lyrics” and “I have ideas for making my work easier” describe them on a 5-point scale (0 = *Never* to 4 = *Daily*).

The Attitudes and Values Scale (α = .75) is a 25-item scale designed to measure attitudes and values related to creativity. Participants endorsed items such as “Sometimes it is best to be unconventional” and “Time is often wasted when everyone involved on a project shares each of his or her ideas (reversed)” on a 5-point scale (1 = *Strongly Disagree*; 5 = *Strongly Agree*).

### Results

We assessed all relationships using zero-order correlations. Education level was unrelated to geek engagement, *r*(394) = -.08, 95% CI [-.18, .01]. Individuals higher in geek engagement showed significantly more creative behavior both in school, *r*(394) = .24, 95% CI [.14,.33], and on their own, *r*(394) = .25, 95% CI [.16,.34], ideational behavior, *r*(394) = .39, 95% CI [.30,.47], and more positive attitudes toward creativity, *r*(394) = .12, 95% CI [.02,.21]. Thus, individuals high in geek engagement report having more ideas, feel compelled to do more creative projects, and value creativity and its products more than individuals low in geek engagement. In addition, individuals who scored higher in geek identity showed significantly more ideational behavior, *r*(394) = .25, 95% CI [.15,.34], positive attitudes toward creativity, *r*(394) = .14, 95% CI [.04,.23], and creative behavior both in school, *r*(394) = .18, 95% CI [.08,.27], and on their own, *r*(394) = .15, 95% CI [.05,.24].

Again, due to some intercorrelation between the creativity indices (*r*s = .03-.33), we ran a separate regression predicting geek engagement with each individual index along with gender, age, SES, and education. All creativity indexes continued to significantly predict geek engagement, while age and social class significantly predicted geek engagement and education marginally predicted geek engagement.

Thus, individuals with high geek engagement not only engaged in opportunities to be creative in work or school, where they may have been encouraged or even made to do so, but also undertook creative endeavors on their own time and of their own accord.

### Discussion

The results of Study 7 provide further support for the desire for engagement hypothesis in that those higher in geek engagement seem to hold positive attitudes toward creativity and engage in more creative activity in general. Identification as a geek was also tied to creative attitudes and behaviors, suggesting that creativity is an acknowledged part of geek culture and considered part of the geek stereotype.

## An Internal Meta-Analysis

**Fig 1:**
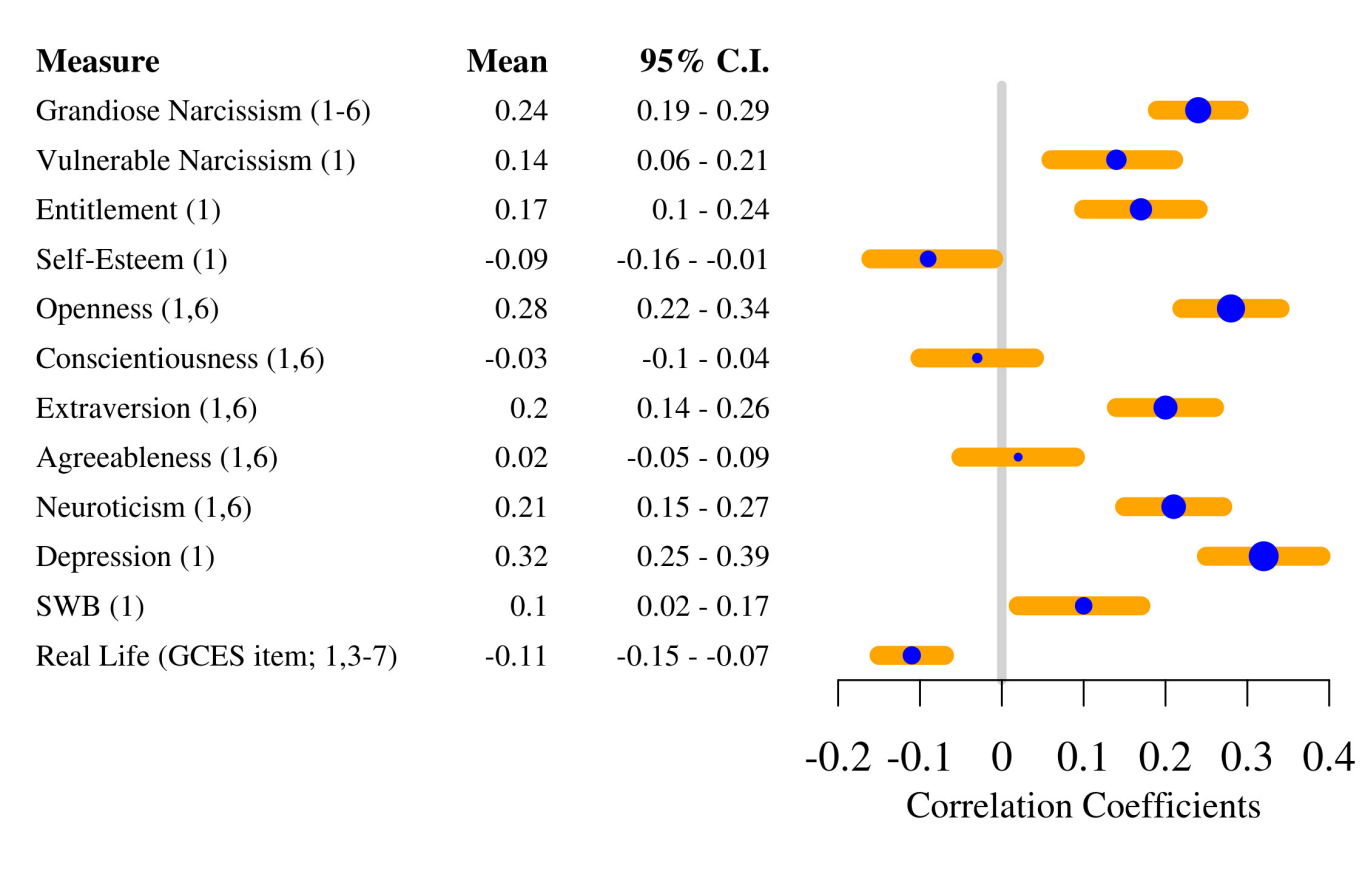
Forest plot of average correlations across studies for selected individual differences variables.

To make the results more clear, we meta-analyzed results collected in more than one sample and present them in Fig 1. Over the course of five studies, narcissism showed a consistently strong positive relationship to geek engagement, *r*(2353) = .24, 95% CI [.19, .29]. This relationship persisted after controlling for age, gender, SES, and in Study 7, education. We can say with high confidence that geek engagement is positively related to narcissism, which provides partial support for the great fantasy migration hypothesis. Hypersensitive narcissism, entitlement, depression and subjective well-being all showed relationships consistently above zero over the course of two studies, whereas the Big Five traits of openness, neuroticism, and extraversion showed average relationships above zero over the course of three studies. However, in Studies 1 and 6, the majority of these relationships went away when controlling for gender age, and SES. Only openness, depression, and SWB maintained significance in Study 1, and openness maintained significance in Study 6 except when fantasy proneness and schizotypal personality were controlled for. The consistency with which these patterns of results appear over multiple studies suggest that the higher a person’s geek engagement, the higher their narcissism,openness, depression, and self-reported subjective well-being. In addition, we meta-analyzed the relationship between the GCES item “real life” and full scale geek engagement as a further test of the great fantasy migration hypothesis. This item showed a consistent negative relationship with geek engagement, suggesting that geeks may perceive themselves as less engaged in their daily lives. However, because we provided little guidance as to definition of real (daily) life, and because of the specificity of geek engagement relative to “real life,” this relationship may also reflect differing definitions of “real life” rather than actual disengagement. Further research is required to determine whether this lack in felt engagement is specific to geek engagement.

## General Discussion

As recently as the 1980’s, comic book heroes, high fantasy, and science fiction—media interests typically associated with geeks—were considered strange, unpopular, and in many cases taboo. In 2014, these same markers of geek culture are box office smashes, multi-billion dollar industries, and a wide-reaching counterculture with its own brands, fashion trends, and celebrities.

We sought to better understand the phenomenon of geek culture primarily at the individual level—that is, to understand why a given individual would choose to engage in geek culture. We developed and validated two scales to measure two major components of geek culture: engagement and identity. We also proposed and found mixed evidence for each of three models of geek cultural engagement. We review these findings below.

### The Geek Culture Engagement Scale (GCES) and the Geek Identity Scale (GIS)

The GCES is the first measure of its kind to focus specifically on the geek subculture. The GCES shows excellent reliability and construct validity. It adequately distinguishes self-identified populations (e.g., Dragon*Con attendees) and correlates positively with actual behavior (Study 3). It captures nuances of geek engagement that are not apparent to naïve observers (Study 2). Despite several of its factors having only two items, it presents a stable factor structure, with the majority of its subscales showing appropriate reliability. The possible exceptions are the Puppetry/Robotics and Roleplaying subscales—despite their face validity, these subscales contain only two items and show relatively low correlations. However, these subscale scores remain correlated to the other subscales and to geek engagement as a whole, and inclusion of their items in the full scale score does little to harm the overall reliability of this measure.

There are important limitations to the interpretations that can be drawn from the GCES. First, because we used major geek conventions to generate the list of activities for the scale, this scale may fail to capture more marginalized geek activities that are not represented at a large convention. Second, because we conceptualized geek culture engagement as involvement in multiple geek activities, this scale may not capture geek “specialists,” or persons engaging intensely in only one geek activity (e.g., an avid Trekkie who only devotes his time to *Star Trek*). Although specialists were relatively rare in our samples, they did appear to differ from other geeks in terms of Big Five personality variables—especially agreeableness, where specialists reported relatively low levels. Thus, the GCES speaks best to generalist geeks, and caution should be used when specifically studying specialist geeks. However, homophily between geeks with specific interests (Study 5) only emerged when controlling for intercorrelation between subscales, and the Geek Identity Scale (GIS) correlates positively with the full scale GCES, implying that identification as a geek intensifies as one is engaged in more and more geek activities. Although geek specialists may exist, these persons may identify less with geek culture per se, and identify more strongly with their chosen fandom, as evidenced by their lower overall score on geek identity.

The GIS also shows excellent internal consistency and reliability across samples as a measure of identification with the geek subculture. Together, these two scales may capture the majority of geek behavior, but more work should be done to measure geek obsessiveness, which may be important in distinguishing higher levels of geek engagement, and more cultural aspects, such as social currency, systems of meaning, and social norms.

We now review the specific hypotheses.

### The “Great Fantasy Migration” Hypothesis

Studies 1, 2, 3, and 6 addressed the great fantasy migration hypothesis, which predicts that individuals high in narcissism and fantasy proneness will engage in the immersive aspects of geek culture at the expense of engagement in real life in order to live out grandiose fantasies. In support of this hypothesis, we find a positive correlation between narcissism and geek engagement, as well as a positive correlation between geek engagement and fantasy proneness (see Fig 1). The item “real life” also shows a consistent inverse relationship with engagement in geek culture. Less consistent with the hypothesis, the results of Study 3 do not support a negative association between geek culture and broader engagement with civic society. Although they showed slight disengagement from political behavior and more trust in the government, those high in geek engagement were more likely to value career, financial, and activist goals, showing greater engagement in their own lives. Thus, we found mixed evidence for the great fantasy migration hypothesis. It may be that narcissistic individuals are indeed engaging more in geek activities, but are still able to remain engaged in their life goals and in civic organizations that are not involved in politics. In a sense, then, geek culture might be an additional outlet for narcissism but not the only one used by individuals. Ultimately, these data provide only a snapshot of geek and real life engagement at one point in time. In order to thoroughly test the great fantasy migration hypothesis, longitudinal data is needed to determine whether depression or negative events at one point in time leads to greater geek engagement at a later point in time, which then leads to reduced engagement in real life events still later. Whether those high in geek engagement also continue to experience the negative effects of failure in real life (e.g., lower self-esteem and subjective well-being) when engaged in geek culture should also be explored. Geek engagement showed a significant positive relationship to depression across several studies after controlling for demographic variables, suggesting that at the time of the survey, at least, individuals high in geek engagement felt depressed.

### The Belongingness Hypothesis

Studies 1, 4, and 5 addressed the belongingness hypothesis, which predicts that individuals will engage in geek culture to fulfill belongingness needs. Belongingness has long been considered a basic need [23] and Self-Determination Theory [24] posits that much of human motivation is driven by basic needs related to belongingness (e.g., relatedness). Study 1 provided little support for this hypothesis, as the relationships subscale of the BPN scale showed no relationship to geek engagement. This implies that those high in geek engagement are neither more nor less likely to have fulfilled their belongingness needs than those who are low in geek engagement. However, in Study 4, whether participants expected to be accepted or rejected by others for participating in geek activities significantly predicted both their self-directed emotions and actual engagement in each activity.

Study 5 addressed the social networking facet of this hypothesis: specifically, individuals engaging in geek activities form ties with others through those activities. Although there was no correlation between geek engagement and network size, density, or centrality, we found distinct evidence of homophily between persons with similar geek engagement scores. This homophily was most pronounced with persons of similar specific geek interests (e.g., Scifi/Fantasy, Roleplaying). While homophily [28] is a well-known phenomenon in multiple domains (e.g. people tend to connect to others who are similar in demographics such as race or SES, personality traits, and beliefs or values), our findings are inconsistent with the stereotype of geeks as loners and social outcasts who engage in geek activities in private [2]. However, whether geeks are finding others with similar interests or introducing their already geeky friends to their specific geek interests can only be tested with a longitudinal study. Another drawback of these data is that they rely on the self-report of a single individual within each network. A social network analysis in which each member of the network provides information on him or herself would be desirable to confirm homophily between geeks.

In sum, those high in geek engagement are more likely to have closer relationships with others who share their specific geek interests, although they are no more likely to have large or dense networks of friends or to be central to their network of friends than those low in geek engagement. They experience positive self-feelings when engaging in geek activities to the extent that they feel those important in their lives will accept them for engaging in these activities, but they do not show thwarted belongingness needs. The results of these three studies can be reconciled if one does not consider geek engagement to be a guaranteed means of fulfilling belongingness needs. Perhaps people engage in geek activities partly with the (conscious or unconscious) goal to achieve belongingness; however, whether they actually succeed may differ depending on the individual. Conversely, fulfilled belongingness may be a byproduct of engaging in geek culture for other reasons, but provides additional reinforcement to continue engaging. Further research that directly assesses the motivations of those engaging in geek culture is needed to fully understand this process.

### The Desire for Engagement Hypothesis

Studies 1, 6 and 7 addressed the desire for engagement hypothesis. This hypothesis suggests that geek engagement will be highest among people who crave emotional and intellectual stimulation, such as those high in openness to experience, creativity, intelligence, need for cognition, and sensation seeking. After controlling for demographic variables, geek engagement was significantly related to openness to experience, fantasy proneness, schizotypal personality and dissociation, all known predictors of creativity. In addition, geek engagement was associated not only with creative behavior, but also ideational behavior and positive attitudes toward creativity, although we did not measure the quality of their creative ideas and products. However, geek engagement showed a negative relationship to crystallized intelligence. This is inconsistent with the commonly held belief that geeks are more intelligent than non-geeks. Overall, although geeks do not appear to particularly need emotional or intellectual stimulation, they require outlets for their creativity, although they may lack intellectual ability and may or may not be creatively talented.

Our predictions were only partially supported with regards to the openness, creativity, and need for stimulation correlated with geek engagement. Of the variables tested, geek engagement appears to be predicted primarily through creativity and its correlates. Neither need for cognition nor sensation seeking appear to play a role. In addition, the negative relationship with crystallized intelligence conflicts with the common belief that geeks are more intelligent than non-geeks. This may partially be a result of using a brief self-report scale of intelligence. Although the Shipley Institute of Living Scale [75] is a well-established brief measure of intelligence, a more in-depth IQ battery such as the WAIS-IV [78] may be needed to detect more nuanced relationships between geek engagement and intelligence. Although surprising, geek engagement’s negative relationship with intelligence coupled with its positive relationships to dissociative and schizotypal symptoms are consistent with DeYoung’s [79] conception of openness to experience as a paradoxical simplex in which intelligence and apophenia (a trait similar to schizotypy) are both related to openness but also different from each other. These results suggest that those who are high in geek engagement are on the apophenia (rather than intelligence) region of the simplex. This could explain the common belief that geeks are more intelligent because their openness resembles that of individuals high in the intellect region of the circumplex. However, their tendency toward apothenia may determine why some individuals high in openness gravitate toward geek activities while others do not.

### A Neuroticism Hypothesis?

We did not predict any relationship with geek engagement and neuroticism or related constructs (e.g., depression) in any of our models. However, the data show a consistent relationship between geek engagement and neuroticism and nonclinical depression as measured by the CES-D (*r*’s around .22). Part of this (as shown in Study 1) might be a consequence of age and gender, because when these were covaried the neuroticism correlations dropped to contain zero in the confidence interval. But there are several other possible explanations. For example, geek engagement could be attractive to people who are depressive because it might serve an emotion regulatory function–basically an escape from unpleasant experiences. Likewise, geek engagement could lead to depression or neuroticism because it isolates one from the mainstream culture and real life. This latter explanation, however, seems unlikely given that our data show geek engagement provides a source of belongingness and does not impair most forms of civic engagement. Also, in Sample B of Study 1, the autonomy subscale of the BPN and the autonomy and impersonal subscales of the GCOS related to geek engagement in such a way as to suggest thwarted autonomy needs and reduced feelings of effectiveness and intrinsic motivation. Individuals who feel ineffective and controlled in real life (and thus may suffer from reduced well-being and depression) may increase their well-being through geek activities that support autonomy. This would be consistent with the leisure coping research more broadly [80]. These findings contrast with the consistent positive relationship between geek engagement and subjective well-being. Clearly, more work needs to be done on these issues–and it may be that these reflect alternate streams of the “great fantasy migration” (i.e., escaping mainstream cultural negativity and autonomy-thwarting environments) or, in the case of neuroticism, an additional motivation for the belongingness hypothesis (i.e., an effort to use geek cultural engagement to reduce neuroticism by increasing belongingness). Research examining whether depression and related constructs are reduced and autonomy is increased during engagement in geek activities can further illuminate this issue.

### Ethical Interpretation of Findings

We are aware that several of our findings have the potential to create or perpetuate social stigma for individuals in geek culture. It is not our intention to link geek culture with dysfunction or antisocial behavior. Although the terms narcissism, fantasy proneness, schizotypy, and dissociation are often used in clinical contexts, the field has moved toward viewing these constructs as dimensional traits, moderate levels of which may be neutral or even adaptive for the individual (e.g., [81]). Narcissism in particular has been studied as an adaptive trait by social psychologists [82,83] and moderate levels of schizotypal personality and dissociation have been shown to be related to creativity [40,41], which can be a form of adaptive functioning. A subfactor resembling schizotypy has been found in the basic personality trait openness to experience [79] and fantasy proneness includes a nonclinical factor that encompasses daydreaming and enjoyment of fantasy [22]. Therefore, relationships between these traits and geek engagement should not be interpreted as evidence of psychopathology in geeks. Individuals high in geek engagement in Studies 1–7 above scored high in all of these traits, but barring some depression, reduced crystalized intelligence, and thwarted autonomy, they also showed increased levels of civic engagement and showed no deficits in belongingness, social network size, or future orientation. Thus we have painted a picture of geeks as different, but not dysfunctional.

## Limitations and Future Directions

In this paper we have only begun to explore the reasons people engage in geek culture. As we state up front, this is a beginning rather than the last word on the topic. We have relied heavily (although not exclusively) on correlational, self-report data to examine the plausibility of the theories posed above. Experimental, developmental or experience sampling methods would be ideal to more definitively test each of the hypotheses proposed in this paper. We have foregone more complex mediational analyses that will eventually be required to provide a definitive test of the mechanisms we have proposed here. We also have not conducted research using other ethusiasts as a comparison group; research comparing geeks to other groups containing like-minded individuals (e.g., football fans) will be needed to determine whether these relationships are exclusive to geeks. Finally, we have focused on these hypotheses at an individual level. Cultural level work exploring major cultural events and demographic information is needed to examine these hypotheses, as geek engagement is a cultural trend as well as an individual behavior.

In addition, there is a strong reliance on MTurk as the source for most of the samples used (with the exception of our sample from Dragon*Con). Although there is little reason to expect MTurkers to differ appreciably from the wider population [42,43], use of a wider range of samples in future work would be useful.

Finally, even within geek engagement, more work needs to be done to discern what makes these media interests part of geek culture. For example, what role does escapism play in geek culture? Is escapism the common factor that attracts geeks to a new franchise? Does the appeal lie in some element of “magic” or controlling the uncontrollable? Do the media need to include some sort of “special” individual who has extraordinary powers or has been chosen for some quest? Additionally, geeks are theorized to share social norms, values, and customs in addition to common interests [6–8]. Work using techniques from cultural psychology or sociology may help to illuminate these elements of geek culture.

## Conclusion

Although it primarily concerns entertainment and leisure, geek culture is becoming an increasingly prevalent part of our society. The study of geek culture can tell us much about how individuals engage with media and for what reasons. Our findings suggest that geek media is especially attractive to narcissists, independent of demographic variables. Given the trend of rising narcissism in the United States [6], understanding geek media may shed light on the function media plays in the narcissistic process. We have also found geek engagement to be related to subclinical depression, making it potentially relevant to clinical psychologists as either a cause or a potential remedy for depressed mood. The GCES and GIS can be used to do important work on each of these social problems.

This paper has taken the first steps toward defining and measuring geek engagement, and has proposed and explored several explanations for its popularity. In addition, this paper takes a unique approach to examining subcultural trends through a personality and individual differences perspective. Much of the past research on group membership has focused on groups in which membership is less freely chosen (e.g., racial and ethnic cultures), but the current research focuses on voluntary participation in a counter culture. This approach has the advantage of targeting in the self-selecting natures of subcultures (for example, how does the greater majority of fantasy prone white males in geek culture affect the norms of that culture?) and may prove helpful in examining future phenomena at both an individual and cultural level. We hope to have laid some useful groundwork for future research exploring such phenomena and their impact on recent generations.

## Appendix

Geek Culture Engagement Scale (GCES)

For each of the following, please indicate to what extent you engage in this activity on a scale from 1 (Not at all) to 5 (A Lot).

Note: Bolded items were retained for the final scale and used in studies 3 through 7. Non-bolded items were only used in study 1, samples A and B and were removed during factor analysis.

**LARPING** (Live Action Role Playing Games)

**Table Top Role Playing Games** (e.g., Dungeons and Dragons, World of Darkness, GURPS)

Computer/Console gaming (World of Warcraft, Half-Life, Minecraft etc.)

**Cosplaying** (making and wearing costumes of Anime characters, superheroes, etc.)

Posting in internet forums (4chan, tumblr, reddit, etc.)

Attending Conventions (Comicon, Dragon Con, etc.)

Attending Renaissance Fairs

The Society for Creative Anachronism (SCA) and other historical reenactments

Weapons Collecting

**Paranormal Investigation** (Ghost Hunting, Psychic Phenomena, reading about the paranormal etc.)

**Puppetry** (making and performing with puppets, muppets, etc.)

**Robotics** (making, using, learning about robots)

**Theater** (acting, costuming, building sets, etc.)

**Creative Writing** (fiction, poetry, etc.)

Social Networking (Facebook, Myspace, etc.)

Your daily life (e.g., work, study, exercise)

For each of the following, please indicate 1) whether or not you are a fan of this genre and 2) to what extent you read/watch/participate in this genre

**Fantasy** (e.g., Lord of the Rings, Harry Potter)

**Sci-Fi** (e.g., Star Trek, Star Wars, Stargate)

**Anime** (Japanese cartoons, e.g., Pokemon, Full Metal Alchemist)

**Manga** (Japanese comic books, e.g., Nana, Fruits Basket)

Comic Books (e.g., Superheroes such as Batman or Superman, V for Vendetta, the Watchmen)

Horror (HP Lovecraft, Korean and Japanese horror, Evil Dead, Stephen King, Anne Rice novels)

**Broadway/Theater/Musicals** (e.g., Phantom of the Opera, Rent)

**Alternative History** (Steampunk, Cyberpunk, retrofuturism, etc.)

Non-Anime Animation (Disney, My Little Pony, Nickelodeon, Cartoon Network)

**British Series** (Sherlock, Dr. Who, Monty Python, Being Human)

**Filking** (Singing minstrel style songs, playing in a band that sings tribute/parody songs about Star Trek, Dr. Who, etc.)

Cinema (Independent Films, etc.)

Joss Whedon Films (Buffy the Vampire Slayer, Angel, Dr. Horrible’s Sing Along Blog, Firefly, etc.)

**Rocky Horror Picture Show** (Live cast performances, props, etc.)

For each of the following, please indicate 1) whether or not you participate in this lifestyle and 2) how often and to what extent you participate in this lifestyle

Skeptic (Freethinkers, science, etc.)

**Lolita** (Japanese fashion)

**Goth/Punk Rock Furry** (anthro, etc.)

**Pagan** (i.e., Wiccan, Norse, etc.)

**BDSM** (Bondage Domination Sadism Masochism)

**Polyamore** (consentual nonmonogamous relationships, having more than one lover, etc.)

Scoring: No items are reverse scored. Full scale is calculated by taking the mean of all bolded items. Subscale scores are calculated by taking the mean of the items in each subscale:

Roleplaying: LARP, Tabletop

Hobbies: Cosplay, Renfaire, SCA, Weapons, Paranormal

Puppetry/Robotics: Puppetry, Robotics

Japanese: Anime, Manga

Genres: Science Fiction, Fantasy, British Series

Theater: Theater, Creative Writing, Broadway, Alternative History, Joss Whedon Films, Rocky Horror

Lifestyles: Lolita, Gothic, Furry, Pagan, BDSM, Polyamore

**Table 10:** Geek Identity Scale (GIS) On a scale from 1 (Strongly Disagree) to 5 (Strongly Agree), please indicate your agreement to the following statements:

	Strongly Disagree			Strongly Agree	
1. I consider myself to be a "geek."	1	2	3	4	5
2. Being a geek is central to my identity.	1	2	3	4	5
3. Being a geek is important to me in my life.	1	2	3	4	5
4. Being a geek is a major part of who I am.	1	2	3	4	5
5. I would describe myself to others as being a geek.	1	2	3	4	5
6. I am proud of being a geek.	1	2	3	4	5
7. If I stopped participating in geek activities, I just wouldn’t be the same person.	1	2	3	4	5
8. I can’t imagine life without my geek interests and activities.	1	2	3	4	5
9. I consider myself to be part of the geek culture.	1	2	3	4	5
10. I value being a geek.	1	2	3	4	5

Scoring: No items are reverse scored. The full scale is calculated by calculating the mean of all items.
